# Exploring the Potential Link between PFAS Exposure and Endometrial Cancer: A Review of Environmental and Sociodemographic Factors

**DOI:** 10.3390/cancers16050983

**Published:** 2024-02-28

**Authors:** Aderonke Ayodele, Emmanuel Obeng-Gyasi

**Affiliations:** 1 Department of Built Environment, North Carolina A&T State University, Greensboro, NC 27411, USA; 2 Environmental Health and Disease Laboratory, North Carolina A&T State University, Greensboro, NC 27411, USA

**Keywords:** PFAS, endometrial cancer, stress

## Abstract

**Simple Summary:**

This exploratory narrative review investigates the association between exposure to per- and polyfluoroalkyl substances (PFAS), sociodemographic factors, stressors, and endometrial cancer risk. It explores the diverse sources of PFAS exposure and examines the role of income, education, occupation, ethnicity, and geographic location in influencing exposure levels and cancer risk. The review finds significant correlations between these sociodemographic factors and both PFAS exposure and endometrial cancer risk. It emphasizes the need for further interdisciplinary research and targeted interventions to understand and address these complex relationships, highlighting the importance of addressing health disparities for effective disease prevention and management.

**Abstract:**

This exploratory narrative review paper delves into the intricate interplay between per- and polyfluoroalkyl substances (PFAS) exposure, sociodemographic factors, and the influence of stressors in the context of endometrial cancer. PFAS, ubiquitous environmental contaminants notorious for their persistence in the ecosystem, have garnered attention for their potential to disrupt endocrine systems and provoke immune responses. We comprehensively examine the various sources of PFAS exposure, encompassing household items, water, air, and soil, thus shedding light on the multifaceted routes through which individuals encounter these compounds. Furthermore, we explore the influence of sociodemographic factors, such as income, education, occupation, ethnicity/race, and geographical location and their relationship to endometrial cancer risk. We also investigated the role of stress on PFAS exposure and endometrial cancer risk. The results revealed a significant impact of sociodemographic factors on both PFAS levels and endometrial cancer risk. Stress emerged as a notable contributing factor influencing PFAS exposure and the development of endometrial cancer, further emphasizing the importance of stress management practices for overall well-being. By synthesizing evidence from diverse fields, this review underscores the need for interdisciplinary research and targeted interventions to comprehensively address the complex relationship between PFAS, sociodemographic factors, stressors, and endometrial cancer.

## 1. Introduction

The intricate interplay between per- and polyfluoroalkyl substances (PFAS) exposure, sociodemographic factors, and the influence of stressors concerning endometrial cancer is a critical issue of public health significance. PFAS, a group of synthetic chemicals widely found in consumer products and industrial processes, has garnered significant attention not only for their persistence in the environment but also for their potential adverse health effects, which have implications for public health [1]. These persistent chemicals, which do not readily break down in the environment, pose challenges by contaminating air, water, and soil, ultimately affecting ecosystems and biodiversity.

Endometrial cancer, the most common gynecologic malignancy in developed countries, has been linked to environmental factors, yet the role of PFAS in its etiology remains underexplored. Recent studies have begun to uncover the extent to which PFAS compounds, known for their ability to disrupt endocrine function and modulate immune response, may contribute to the pathogenesis of endometrial cancer [2,3]. The exposure to these substances is not uniform, varying significantly across different sociodemographic groups. Factors such as socioeconomic status, education level, occupation, ethnicity, and geographic location play a crucial role in determining the degree and type of PFAS exposure. For instance, industrial settings often present higher environmental concentrations of PFAS, impacting local populations disproportionately. Thus, investigating the association between PFAS exposure and EC is timely and important due to emerging evidence, public health implications, environmental concerns, and clinical relevance [2,4]. Addressing this research gap can contribute to our understanding of environmental carcinogenesis and inform strategies to mitigate the health risks associated with PFAS exposure.

Moreover, the influence of psychosocial and environmental stressors adds another layer of complexity to this relationship [5,6]. Stress has been implicated in various disease processes through mechanisms such as the activation of the hypothalamic–pituitary–adrenal (HPA) axis and subsequent alteration of hormonal and immune responses. In the context of endometrial cancer, stress may act as a modulator, influencing how the body responds to environmental toxins like PFAS [7,8]. The concept of allostatic load, which represents the cumulative burden of chronic stress and life events, is particularly relevant here. It is hypothesized that individuals with higher allostatic load may exhibit increased sensitivity to the harmful effects of PFAS, thereby elevating their risk for endometrial cancer [9,10,11].

Recent scientific investigations have established a correlation between exposure to certain PFAS and an elevated risk of specific cancer types [12]. Notably, PFOA has been implicated in heightened incidences of kidney and testicular cancers [13]. The exploration into PFAS’s association with other cancer forms, including endometrial cancer, remains ongoing and an area of active research [14].

The oncogenic potential of PFAS is hypothesized to be mediated through a spectrum of biological mechanisms. These encompass hormonal disruption, immunotoxic effects, induction of oxidative stress, and perturbation of cellular signaling pathways [15,16]. PFAS have the capacity to bind to hormone receptors, modulate gene expression, and disrupt endocrine functions, potentially culminating in carcinogenesis [17].

In addition to these mechanisms, PFAS are known to exert epigenetic effects, notably the alteration of gene expression independent of DNA sequence modifications [18]. Epigenetic regulation is instrumental in controlling cellular processes such as growth, differentiation, and apoptosis, all of which are pivotal in cancer development [19]. Exposure to PFAS has been associated with variations in DNA methylation patterns and histone modifications [20]. These epigenetic alterations have the potential to activate oncogenes or silence tumor suppressor genes, thus contributing to the onset and progression of cancer.

Beyond their carcinogenic implications, PFAS also exhibit broader health impacts. They are recognized for their endocrine-disrupting properties, which have extensive repercussions on human health, including effects on reproductive health, thyroid function, and an increased risk of hormone-related cancers. Furthermore, PFAS exposure has been linked to alterations in immune function. This can not only contribute to the emergence of cancer but also impair the body’s capacity to combat infections and other diseases. Of particular concern is the exposure of pregnant women and children to PFAS. These compounds can traverse the placental barrier and impact fetal development, potentially leading to persistent health issues [21].

This paper aims to assess the relationship between each sociodemographic variable (such as income, education, occupation, zip code or geographical location, and ethnicity), the exposure (PFAS), and outcome (EC). This paper is unique in the context of the current literature because it considers this relationship in the context of sociodemographic factors and stressors when exploring the relationship between PFAS and EC. The review article is also meant to explore potential associations between PFAS exposure and risk of endometrial cancer, and the purpose of the article is not about establishing causality in PFAS exposure. By integrating findings from various studies, we seek to delineate the complex pathways through which these elements interact, contributing to the incidence and progression of this disease. Understanding these interconnections is crucial for developing targeted public health strategies and interventions to mitigate the risk and burden of endometrial cancer, particularly in populations most vulnerable to PFAS exposure and stress-related impacts. It must be noted that this exploratory review aims to generate hypotheses and encourage further research rather than assert a definitive causal relationship. Our review of both toxicological and epidemiological evidence helps to understand both correlational analysis and biological plausibility.

### 1.1. What Are Per- and Polyfluoroalkyl Substances (PFAS)?

PFAS are amphiphilic organic substances that have been used in several industrial applications, such as firefighting materials and protective coatings [22]. They are environmentally persistent and are generally highly resistant to any form of degradation (such as hydrolysis, photolysis, and metabolism) due to the polar covalent bond between carbon and fluorine atoms [23,24]. Due to their persistency, PFAS possess varying half-lives in biological samples [25], with long-chain PFAS having half-lives ranging from 2.86 to 3 years [26]. The long decay time is the primary reason PFAS have bioaccumulated in terrestrial and aquatic ecosystems [27]. Due to the human food chain model, PFAS are either ingested through drinking contaminated water or animal/plant products [28]. In the United States, serum concentrations of different classes of PFAS were detected in about 98% of the adult population between 2003 and 2014 [29]. However, based on the adverse health effects of certain PFAS, especially PFOA and PFOS, they are being phased out in the United States [2,30].

To reduce the environmental burden of PFAS, a stringent policy on prohibition and complete removal of contaminated materials needs to be enacted into law. There are many scientific reports utilizing various adsorbents in PFAS-contaminated sites [31,32]. An in situ detection, removal and destruction of PFAS in water looks attractive and could pave the way forward for reduced environmental contamination [33]. However, removal efficiencies of PFAS in various environmental media (such as soil, air or water) could be daunting due to varying chemical interactions [34].

Prolonged exposure to PFAS is also known to cause other health problems [35]. However, the main challenges are the fact that they are found in so many household items and drinking water. There are different types of PFAS on the global market [28,36,37,38,39,40], while only ~28 PFAS are quantitatively analyzed and identified [22]. Controlling exposure to PFAS can quickly be completed when the unknown PFAS are identified, and exposure assessment can limit or prevent future routes of administration. Prior research has found that factors such as income, race and ethnicity, marital status, and age play a significant role in determining PFAS exposure among women in the United States [41,42]. Therefore, the purpose of this review is to understand the connection and variability between the PFAS route of administration and sociodemographic attributes. Additionally, a detailed review on the toxicity and occurrence of PFAS and its connection to endometrial cancer will give insight into the molecular and cellular mechanisms that lead to the development of endometrial tumors.

#### 1.1.1. PFAS in Household Items

The addition of PFAS to consumer products has been practiced since the 1940s in the United States (U.S.) [43]. For instance, the ability of PFAS to confer non-stick, grease, and oil-resistant properties in cookware has influenced their adoption in kitchenware industries [44,45]. These anthropogenic chemicals are also used to produce other household items, such as stain-resistant furniture and carpets [1], fire extinguishers, water-repellent raincoats, and grease-resistant containers [46]. Commonly found PFAS in household items are perfluoroalkane sulfonic acids (PFSAs), perfluoroalkyl carboxylic acids (PFCAs), fluorotelomer alcohols (FTOHs), perfluoroalkane sulfonamides (FASAs) [44,47]. However, the addition of PFAS to household items mostly is not disclosed on either the label or product information, according to a recent study [44]. A similar study found PFAS in North American school uniforms marketed as stain-resistant textiles [48]. The prevalence of PFAS in household items in North America and the United States is a serious concern, as a 2012 study revealed elevated serum levels of PFAS in individuals exposed to these items [49]. In a bid to determine the association between elevated PFAS serum levels and household items, Zhu et al. [1] monitored the serum concentrations of PFAS in the general US population aged 12 years and older using the 2005–2006 National Health and Nutrition Examination Survey (NHANES) and found out that individuals in homes with low pile carpet (carpet with taller and looser fibers) usage have considerably higher concentrations of perfluorohexane sulfonic acid (PFHxS) and 2-N-methyl-perfluorooctane sulfonamido) acetic acid (MeFOSAA) when compared with people residing in homes with smooth surfaces. Other studies corroborate the association between elevated PFAS exposure and commodity products [50,51,52,53].

#### 1.1.2. PFAS in Water

Contamination of U.S. water bodies with PFAS is a significant health concern for people close to water sources near an industry with a legacy of PFAS production. Previously, the lifetime health advisory level for PFAS of 70 ng/L in drinking water, especially for PFOS and PFOA, was submitted in 2016 [54,55]. However, the concentration of PFAS detected in the U.S. water systems of selected large communities was still high, despite testing a limited number of PFAS and omitting sampling of water systems in smaller communities [56]. This issue led the EPA to propose a National Primary Drinking Water Regulation (NPDWR) to legally establish enforceable levels of ~4.0 ng/L PFAS in drinking water from the year 2023 [57].

Using a small remote community in Alaska (Gustavus) as a case study, Babayev et al. assessed PFAS exposure and contamination of drinking water. They concluded that significant PFAS sources include airport operations and fire training sites [58,59,60]. The detection of PFOA and PFOS has been reported in the literature [61,62,63], but most recently, other types of PFAS such as fluoroethers, GenX, perfluorohexane sulfonic acid (PFHxS), PFNA, perfluorobutyric acid (PFBA) [64,65,66]. For example, 2,3,3,3,-tetrafluoro-2-(1,1,2,2,3,3,3-heptafluoropropoxy)-propanoate (GenX) and other PFAS were detected in the Cape Fear River located around a PFAS chemical manufacturing industry in Fayetteville, North Carolina [67], and about 837 private wells within a 5 mile radius of the industry revealed approximately 25% (207) of the wells were contaminated with GenX [68].

The disease burden of water-contaminated wells with PFAS are rather inconclusive, but several studies have linked prolonged PFAS exposure to low birthweight in children born to exposed mothers [69,70,71,72]. Continued contamination of surface and underground water in the U.S. is worrisome, and PFAS concentration benchmark (mainly PFOA and PFOS) set by U.S. EPA has been deemed not sufficiently protective by scientists and state health agencies [e.g., North Carolina Department of Health and Human Services (NCDHHS)] [59,73,74]. In order to lower PFAS concentrations in water, about nine states in the U.S., including Massachusetts, Vermont, and New Hampshire, have placed higher stringent guidelines limiting the total concentrations of about six PFAS to between 8 and 20 nanograms per liter (ng/L) for drinking water [55,74].

#### 1.1.3. PFAS in Soil

PFAS contamination of soil can occur through human and industrial activities, and one active pathway of PFAS contamination is via the use of aqueous film forming foam (AFFF) in fire extinguishers [75,76]. Indirect contamination also occurs through the transportation of PFAS from household items, wastewater treatment plants, fertilizers, biosolids, and landfill leachates [75,77]. In one study, a detailed mapping of a Norwegian firefighting training facility revealed a high concentration of PFAS, with PFOS accounting for about 96% of the total PFAS found in the soil [78]. However, AFFF-contaminated sites appear to be active PFAS reservoirs, mainly due to PFAS mobility in saturated subsurface soils [79].

Although short-chain PFAS migrate into groundwater, the long-chain PFAS are readily retained on the soil surface and can easily be transported through ecological receptors [80]. Most recently, the application of digested sewage sludge (biosolids) to soils has been implicated as a significant source of PFAS’s contamination of surface soils, deeper soils (vadose zone), and the underlying groundwater [81,82]. The most found PFAS in biosolids are PFOS, PFDA, and PFOA. They can bioaccumulate in agricultural products, thus presenting a feasible pathway for trophic circulation of PFAS within the food chains which could lead to severe human and animal health issues [83,84]. Jha and colleagues reviewed a scenario of an integrated crop-livestock system practiced on a facility close to a firefighting site. The study concluded that PFAS was found in the nearby groundwater. Animal milk resulting from the irrigation of crops or management of livestock from contaminated surface/groundwater contained significant PFAS [85]. PFAS-laden milk/meat product consumption harms human health [86]. Phytoremediation with plants is a promising strategy for limiting PFAS in contaminated soils [87,88].

#### 1.1.4. PFAS in the Air

The need to study the accumulation of PFAS in air was borne out of the extensive studies on the recalcitrance nature of the ‘forever chemical’ in water and soil [89,90,91] and its impact on human health [92]; however, the extent of direct human exposure to airborne PFAS through inhalation or other uptake mechanisms remains largely unknown [93,94]. Although the persistence of PFAS or their terminal perfluorinated degradation products is due primarily to their strong C–F bond [95,96], their local and long-term accumulation in air is difficult [93]. Therefore, the atmosphere only provides a route for long-range transport of PFAS, resulting in low concentrations in a localized point of PFAS contamination site [97] which has necessitated most air studies to determine PFAS concentrations in surrounding contaminated sites [98,99].

Chemours Company is a domestic producer of perfluoroalkoxy alkanes (PFA), a type of fluoropolymer that is similar in chemistry to PFAS and degrades to PFAS as a byproduct [100]. Likewise, their water and air emission patterns are associated with the method of production, use, and disposal [100]. Therefore, using the Chemours Company Fayetteville Works Plant as a case study, regarding 2015 and 2016 publications, the discovery of HFPO-DA contamination of the Cape Fear River and the surrounding soil and surface water, located about 25 km away from the company [101,102], could be attributed to air transport and deposition phenomena [103,104]. In a bid to fully understand the atmospheric transport and fate of PFAS, D’Ambro and colleagues modelled the air quality of a fluoropolymer manufacturing facility in North Carolina using the Community Multiscale Air Quality (CMAQ) model at high resolution of within 1–150 km from the facility [93]. The model predicted a total emission of about 5% PFAS and 2.5% GenX which were detected about 150 km of the facility and the modeled air concentrations of these legacy chemicals fluctuated based on wind speed [93].

Similarly, previous studies on the pervasive presence of PFCA, diPAPs, and POSF-based materials in indoor dust or air [105,106] were due to some consumer products, such as carpets, and an elevated PFAS serum level found in children aged 12 years and older was associated with consistent usage of stain-resistant carpets [1].

#### 1.1.5. Importance of Studying the Relationship between PFAS and Endometrial Cancer

Investigating the relationship between PFAS and Endometrial Cancer holds paramount importance within public health research. PFAS exhibits bioaccumulative tendencies and environmental persistence. Scientific evidence has indicated a plausible association between them. Thoroughly scrutinizing the relationship between PFAS exposure and endometrial cancer risk is imperative for comprehensive risk assessment, regulatory formulation, and preventive measures. Such endeavors are pivotal in elucidating the mechanisms of carcinogenesis, shaping public health policies, and devising interventions aimed at mitigating PFAS exposure, thereby potentially curtailing the incidence of endometrial cancer.

### 1.2. What Is Endometrial Cancer

#### 1.2.1. Epidemiology of Endometrial Cancer

Endometrial cancer (EC) is a tumor found in the endometrium, and the most occurring histological subtype, endometrioid adenocarcinoma, constitutes about 75–80% of all cases of EC according to studies published in 2009 and 2016 [107,108]. According to 2012 data, this gynecological disease is mostly prevalent in developed countries [109], and it is symptomatic at an early stage, which helps in early diagnosis and treatment [108]. Despite the early diagnosis, in a 2019 study using 2012 data, about 319,000 new cases were reported with 76,160 deaths [110]. This incidence has been associated mainly to lifestyle factors such as diabetes, obesity, age (adults), and socioeconomic factors [111]. EC is primarily observed in women after menopause, with more than 90% of cases occurring in women over the age of 50 [112]. Nevertheless, the growing incidence of obesity may contribute to a surge in the proportion of premenopausal cases, according to a 2016 article [113].

Endometrial cancer is classified into two types: Type I and Type II. Type I tumors are primarily endometrioid and linked to estrogen exposure, accounting for 80–90% of cases but only 40% of the mortality according to studies published in 2013 [114,115]. On the other hand, Type II tumors, including serous and clear cell cancers, have a high fatality rate [116]. To significantly impact mortality, early detection and prevention strategies should concentrate on detecting Type II cancers and identifying fatal cases, regardless of type [112].

A 2016 study suggests that EC has four distinct molecular subtypes, namely mismatch repair (MMR) deficient (deficient for MSH6 and PMS2), POLE exonuclease domain mutated (POLE EDM), p53 wild-type, and p53 abnormal (null or missense mutations) [117].

Although most cases of endometrial cancer have been reported in the developed countries, it is projected that EC incidence will increase among low- and middle-income countries [110]. Brüggmann and colleagues documented the number of EC-related studies, with USA, China and Greece having the highest number of studies while the density mapping indicated that large parts of Asia, Africa, and South America with a high burden of EC have almost no research studies on the disease [118]. Despite being the leading country in EC-related research, USA continues to have the highest number of incident cases (60,000) and 10,000 deaths each year [119]. The study further projected that the EC incidence in the US will rise to 120,000 cases by 2030 which could make the disease become the third most prevalent cancer in women living in the US [119]. Understanding the risk factors and implementing early and accurate detection strategies are crucial to alleviate the health burden of EC.

Endometrial carcinoma is considered to have a positive prognosis since most cases are limited to the uterus and can be effectively treated [120]. The 5 year survival rates for endometrial cancer, broken down by stage, are as follows: 80% for stage I, 60% for stage II, 30% for stage III, and 5% for stage IV according to studies published in 1997 and 2001 [121,122]. The treatment of EC can range from hysterectomy surgery, radiation therapy and chemotherapy for early stages to palliative treatment such as chemo, hormone, or targeted therapies for advanced stage. Overall, 5 year survival rates have shown a consistent gap between black women (approximately 55%) and white women (approximately 85%) from the mid-1970s to the late 1990s [123]. The racial disparities in 5 year survival rates between EC patients can be attributed to a complex interplay of various factors, including healthcare access, socioeconomic status, biological differences, and healthcare disparities. However, this cannot account for the entire difference, as black women still exhibit lower survival rates within specific stages, indicating more advanced disease within the stage, less favorable histological types, or inferior access to quality medical care after diagnosis [121,123].

EC is often detected at an early stage, with vaginal bleeding serving as an early warning sign in 80% of cases [107,124]. The primary courses of action are surgery and radiation therapy, with the cornerstone of treatment being total abdominal hysterectomy and bilateral salpingo-oophorectomy, combined with lymph node dissection. For patients with Stage IC or stage IA and IB with grade 2 or 3 histology, and the presence of adverse risk factors like lymphovascular space invasion, advanced age, tumor size, and lower uterine segment involvement, it is recommended to undergo vaginal brachytherapy and pelvic radiotherapy to reduce the risk of pelvic recurrence according to studies published in 2004 and 2006 [125,126]. Although postoperative radiation therapy can enhance local control, it has no impact on survival in patients with Stage I endometrial cancer. Women with disseminated disease or extrapelvic recurrence are typically reserved for systemic chemotherapy. While cisplatin and doxorubicin combination are frequently used, carboplatin and paclitaxel are highly effective, low-toxicity alternatives for advanced or recurrent cases [127,128].

As stated previously, the occurrence of EC in women has been attributed to some epidemiolocal and lifestyle factors. For example, obesity has been observed as a contributory factor to about 30–40% cases of EC [112,129] due to its estrogenic effects on adipose tissue, thereby causing the proliferation of the endometrium [112].

Moreover, an increased body adiposity index (BAI) has been found to raise the risk of cancer-related mortality [130]. A review of literature conducted by the International Agency for Research on Cancer (IARC) has established a link between BAI and endometrial cancer. The review further explains that a higher BMI accounts for 35% of endometrial cancer cases [131], which further supports evidence of a dose–response relationship between obesity and EC as indicated in a 2015 review [132]. In a review conducted by MacKintosh and Crosbie in 2013, the inter-dependency of obesity and EC was confirmed. It was found that women who were previously obese experienced a reduced risk of EC after losing weight [133]. In addition, in a retrospective cohort study from 1984 to 2002, obese women who underwent bariatric surgery experienced a 78% reduction in the risk of EC even after years of follow-up, according to research by Adams and colleagues [134].

Another important risk factor is polycystic ovarian syndrome (PCOS), a diverse group of medical conditions that are distinguished by the presence of polyfollicular ovaries and an increase in androgen secretion that is dependent on LH (luteinizing hormone). Thus, it has been reported that women with PCOS have higher chances (about 2.7-fold) of developing EC over their lifetime [135,136]. In a recent study, the potential stimulatory effects of serum exosomal miR-27a-5p found in patients with PCOS was explored on EC cell lines. The researchers found that the EC cell lines promoted a more pronounced migration and invasion phenotype [137]. Endogenous and exogenous hormones are also known to be risk factors in women developing EC, with previous data affirming that women with active uterus taking Hormone Replacement Therapy (HRT), e.g., estrogen, had a 2.3-fold increased risk of developing EC [112,138]. There are other risk factors for EC such as diabetes and cardiovascular disease [139,140], tamoxifen [141,142] and other lifestyle factors have also been linked to the development of EC in postmenopausal women [143,144,145].

#### 1.2.2. Endometrial Cancer in the United States

In recent years, there has been a notable rise in endometrial cancer cases, potentially linked to a decrease in hysterectomies performed for benign reasons [146,147]. From 2013 onwards, reports have indicated approximately 49,500 documented cases of endometrial cancer, resulting in around 8200 deaths [146,148]. This upward trend in new cases indicates a growing significance of this disease over the last decade. For instance, in 2012, it ranked as the fourth most prevalent cancer among women in the United States [149,150].

The National Cancer Institute’s Surveillance, Epidemiology, and End Result (SEER) Program collects and publishes cancer incidence and survival data from population-based cancer registries, representing approximately 28% of the U.S population [150]. Within the SEER data, there are varying trends in the increase in endometrial cancer incidence over time, with particularly notable rises observed in black women compared to white women [150]. 

A consistent increase in incidence among women aged 50–74 years, with annual percentage changes ranging from 2.8% to 4.2% during specific time periods, was reported by Trabert and colleagues [151], and these increases were observed also in Women’s Health Initiative (WHI) data [152]. Some of the risk factors of EC concerning sociodemographic variables will be discussed fully in the results. However, it is imperative to note that EC’s prognosis is mainly determined by the tumor with respect to its stages and histological features [153].

## 2. Materials and Methods

This narrative review was conducted to explore the complex interplay between per- and polyfluoroalkyl substances (PFAS) exposure, sociodemographic factors, stressors, and the risk of endometrial cancer. Our approach involved a comprehensive literature search across PubMed, Scopus, Web of Science, and Google Scholar databases. The search utilized keywords including “PFAS”, “endometrial cancer”, “sociodemographic factors”, “stressors”, “environmental exposure”, and “allostatic load”, combined using Boolean operators. We excluded non-peer-reviewed articles, conference abstracts, and publications outside the scope of this study. Data extraction was performed independently by two reviewers who focused on study design, sources of PFAS exposure, sociodemographic variables, stressors, and endometrial cancer outcomes. The narrative synthesis method was employed to combine these findings. Special attention was given to sociodemographic factors like income, education, occupation, ethnicity/race, and geographical location, including radiation, and their roles in modulating PFAS exposure and endometrial cancer risk. In this study, race was included as a variable because, in the context of this study, race is a proxy for various factors, including genetic predispositions, environmental exposures, and social determinants of health. While the study primarily focuses on genetic risk factors, race can also serve as an indicator of environmental injustice. This includes differential exposure to pollutants, including PFAS, due to socioeconomic factors and geographical disparities.

We further conducted a comparative analysis to discern patterns and disparities in these factors across different studies. The review also delved into identifying various psychosocial and environmental stress from the literature, examining their potential roles in mediating the relationship between PFAS exposure and cancer risk.

Quality assessment of each study was rigorously conducted. Any discrepancies in quality assessment between reviewers were resolved through discussion and consensus. Our integrative narrative approach allowed us to combine environmental science, epidemiology, and psychosocial research findings, identifying major themes and trends within existing literature. Ethical considerations, such as proper citation and acknowledgment of sources, were strictly adhered to throughout the review process. Through this comprehensive methodology, this review provides a nuanced understanding of the intersection between PFAS exposure, sociodemographic factors, and stressors in the context of endometrial cancer, thereby guiding future research and public health interventions.

## 3. Results

### 3.1. Sociodemographic Variables

The sociodemographic variables investigated in relation to both PFAS exposure and endometrial cancer are summarized as follows: income, education, occupation, zip code or geographical location, and ethnicity. Each of these factors plays a pivotal role in determining the extent and nature of exposure to PFAS, as well as the associated risk of developing endometrial cancer. Income level, for instance, can influence an individual’s living conditions and access to healthcare, affecting both exposure and disease outcomes.

#### 3.1.1. PFAS

Exposure of humans to PFAS could depend on sociodemographic metrics such as income, ethnicity or education. Some of the factors that will be discussed further in this manuscript are presented in Table 1 which summarizes critical sociodemographic factors stratified over four PFAS in National Health and Nutrition Examination Survey (NHANES) 2003–2006 data as this data has continued relevance.

##### Income

Income has been known to play a role in human exposure to ‘forever chemicals’, as higher income earners are exposed to PFAS usually found in high-end products. Invariably, maternal serum PFAS was associated with some demographic factors including income and researchers observed that PFOA, PFOS, PFNA and PFHxS concentrations were lower among non-Hispanic black women and women with low household income than non-Hispanic white women and high-income earners with recent studies confirming the relationship between PFAS and income [155,156]. In a 2018 research report by Kingsley and colleagues, linear regression was used to determine the relationship between gestational natural log-transformed serum PFAS concentrations and maternal factors such as household income [157]. They observed that household income is related to higher gestational serum PFAS concentrations, especially with PFOS, and their conclusions agree with previous studies [155].

Further analysis of data from 1999 to 2008 NHANES revealed that non-Hispanic black females (less affluent population) had much lower exposure to PFOA, PFOS and PFHxS when compared with non-Hispanic white females (more affluent population) [158,159]. The reason for these differences is presumed to be due to either affluent lifestyle (ability to afford more PFAS-containing products), type of diet, or a combination of both [158]; that said, this study was conducted among a group with data from 1999 to 2008, thus the findings may not apply today. However, in a population of non-Hispanic black men and non-Hispanic white men, there was no significant difference in PFAS concentrations under the household income category [160]. This outcome might be due to shared exposure sources, similar environmental contexts, and potential systemic factors beyond income, affecting both racial groups similarly despite income variations. Another study by Chang et al. among pregnant African American women found a positive but non-significant relationship between poverty income ratio and serum PFAS concentrations [161], while Nelson et al. observed a corresponding increase in human concentrations of PFOS and PFOA with increasing income especially among non-Hispanic white people [154]. The correlation between higher income levels and increased PFOS and PFOA concentrations among non-Hispanic white individuals suggests that lifestyle choices, purchasing behaviors, and potential occupational exposures associated with higher incomes might contribute to elevated PFAS exposure in this demographic group. The work by Nelson et al. occurred much earlier than Chang et al.’s work, though.

#### 3.1.2. Education

The inter-relationships between serum PFAS concentrations and level of education have been partly studied, with a study conducted in the United States affirming that high PFAS concentrations were observed among women with a higher education, irrespective of their marital or birthing status [162]. Conversely, in another population, serum levels of PFOA, PFOS, PFNA and PFHxS were higher in women with a college or graduate education and higher with women who were married or co-habiting with a partner [162]. A correlation (using fit linear regression modeling) carried out by Wise and colleagues on the plasma concentrations of PFAS among reproductive-aged black women revealed a positive association of some PFAS (such as PFDA and PFUnDA) with the level of education. However, the level of education was negatively associated with MeFOSAA, PFHxS, PFNA, or PFOA [163].

Similarly, in a pregnant African American women population within the Atlanta, Georgia region, higher PFNA concentrations were directly related to higher education. The sampled population with college and university degrees had a 57.8% increase in serum PFNA level compared to the group with lesser education [161]. However, PFAS concentrations, in a study conducted between 1999 and 2002, were found to be lowered in a population with higher educational achievement using data subjected to a multivariable linear regression model [164].

PFAS, being a notable environmental concern, intersects with maternal education and the diagnosis of Autism Spectrum Disorder (ASD) and Attention-Deficit Hyperactivity Disorder (ADHD) in children [165,166]. Studies suggest that lower maternal educational levels correlate with ADHD symptoms and associated academic challenges in children, whereas higher-than-average maternal education, like a high school graduate or college degree, tends to be linked with an increased prevalence of autism diagnosis [165,166]. The reasoning behind the higher occurrence of ASD diagnosis in children among mothers with higher educational attainment potentially relates to their elevated socioeconomic status (SES) [167,168,169]. A 2010 study found that this higher SES allows educated pregnant women to consume more fish and seafood [170], known as potential sources of PFAS exposure, possibly contributing to the observed correlation between maternal education, ASD diagnosis, and PFAS exposure.

Another interesting study examined, among other factors, the influence of education on vegans as compared to omnivorous diet in a sample population exposed to various PFAS. The authors found that there were no significant differences in education among this PFAS exposed population (*p*-value of 0.6) when vegans were compared to omnivores [171].

#### 3.1.3. Occupation

Occupational PFAS exposure is of major concern to workers working in fields where large amounts of PFAS is used routinely for various applications. Some of the health risks associated with PFAS exposure are thyroid disruption, cancer and neurological effects [86]. Large exposure of PFAS to susceptible workers are not only limited to fluorochemical plant workers but firefighting, skiing waxing, and textile workers, are also at a risk of being exposed [172,173]. Workers are usually exposed to PFAS in the form of aerosols or volatile matters during the production of PFAS-laden products [174].

Firefighting services represent a commonly studied occupation closely associated with PFAS due to the use of PFAS-containing firefighting foams. Among these foams are Aqueous Film-Forming Foams (AFFF), as well as alcohol-resistant AFFF (AR-AFFF) and protein foams, making firefighting services one of the most extensively researched occupations in relation to PFAS exposure [45,52,174,175]. A study published in 2021 revealed that even in volunteer firefighters, the serum PFAS concentrations of PFDoA, PFNA and PFDA were markedly elevated when compared with NHANES, and the serum levels of both PFDA and PFDoA were directly related to their years of experience in firefighting [52]. A comprehensive study considered two firefighters (FFs) at two different locations in Ohio, United States: (1) airport, and (2) suburban, and the impact of PFAS on their metabolic syndrome (MetS) profile was investigated [176]. The researchers noted higher PFAS serum concentrations in firefighters compared to the general population, with elevated levels ranging from 21% to 62% in airport firefighters compared to suburban firefighters. However, no significant association was found between PFAS exposure and metabolic syndrome (MetS).

It is well documented that AFFF is a significant source of serum PFAS in FFs [82,172,173]; however, FF textiles represent a potent source of PFAS due to the fluoropolymers in FF textiles and when leached out, the PFAS are more mobile leading to a significant exposure source for FF [175]. Firefighting services have been known as a major source of PFAS and the literature abounds on this subject matter; unfortunately, the same cannot be said of other occupations that are known to contribute to environmental PFAS pollution [173]. In studies published in 2010, researchers from Scandinavian countries investigated the serum concentrations of per- and polyfluoroalkyl substances (PFAS) among professional ski waxers. They found that PFOA and other PFAS bioaccumulated in these individuals, leading to higher levels compared to the general population [177,178]. In the U.S., Crawford and colleagues only conducted electronic survey to evaluate risk of PFAS exposure through the application of fluorinated wax frequently used by professional skiers [179], but they failed to analyze the type of PFAS and the concentrations the skiers were exposed to annually.

#### 3.1.4. Zip Code or Geographical Location

The longitudinal trend of PFAS serum levels has sometimes been influenced by residential location, and as such, tracking PFAS exposure through zip code or geographical location is as important as providing answers to the source of PFAS contamination. A cohort study of individuals along the same zip-code over a period of time is assumed to be ideal, and a reduction in exposure variation can be observed in a study population, as was carried out in a 2012 study [180]. However, Stubleski et al. [181] noted that the correlation of a longitudinal study in PFAS serum concentrations in a large population has rarely been studied. Additionally, ref. [180] was conducted between 1996 and 2010, speaking to the dearth of research in this area. Using this approach, neonates’ exposure to PFAS from 2002 to 2011 within the east Minneapolis—St. Paul Metropolitan area was monitored based on the zip code/geographic location demographics [182].

Petriello and colleagues examined the ability of PFAS to interfere with cholesterol in individuals on lifestyle-based lipid-lowering interventions [183]. They quantified six PFAS (namely: PFHxS, PFOS, PFOA, PFNA, PFHpA and PFBS) in 350 individuals and the candidate’s exposure to PFAS was assessed using spatial explanatory variables using the Exploratory Regression tool in ArcGIS Pro with the PFAS as the dependent variable, and some sociodemographic information at the zip code/geographical location level were included in the model. Using the GIS approach, the researchers were able to correlate the geographical location/zip code information collected at baseline to determine PFAS concentrations pre- or post-intervention, and a case study of elevated plasma PFNA levels was observed in Western Kentucky in the zip codes/geographical locations around the Madisonville border [183].

The association of serum PFAS levels in people living around a contaminated public water system with the area’s zip code/geographical location was examined in Californian women [184]. Another study selected participants that had been exposed to PFAS, based on living in zip codes/geographical locations in proximity to the Ohio River sometime after 1980 [185]. Likewise, the association of one or more PFAS in the water systems of North Carolina residents with chronic disease and multimorbidity was assessed based on their zip codes/geographical locations [186]. The effective risk management strategy for PFAS contamination is on-time communication. A study buttressed the importance of using a short message technique to inform participants of exposure to a high concentration of PFAS in their drinking water based on their zip codes/geographical locations [187].

#### 3.1.5. Ethnicity

The role of ethnicity in PFAS exposure is gaining much-needed attention, and particularly there is an association between higher PFAS, blood pressure and the development of hypertension among midlife black women [188,189,190]. Although the drivers of racial/ethnic differences in PFAS exposure have not been well studied, it is possible that such differences could be attributed to residential racial segregation. This is because residing in areas supplied with PFAS-contaminated water has been found to correlate with higher levels of some PFAS in the serum [190]. The motivation to study human exposure to PFAS in relation to ethnicity is on the increase, as some studies have observed racial disparities in PFAS exposure.

In our previous study, it was observed that PFAS serum levels among older adults were influenced by ethnicity/race and the highest concentrations were found among non-Hispanic blacks [160]. A comprehensive study of 1302 women aged 45–56 years, and from different ethnicities and origins (black from Southeast Michigan, Pittsburgh, Boston; Chinese from Oakland; Japanese from Los Angeles) was carried out [42]. The sampling of participants’ PFAS serum was conducted from 1999 to 2000, and linear regression with backward elimination ensured the identification of important parameters of PFAS serum concentrations within a pre-specified variable. It was discovered that white women had a higher serum concentration of PFOA while black people had a higher concentration of PFOS and 2-(N-methyl-perfluorooctane sulfonamide) acetic acid, and Chinese and Japanese people had elevated levels of PFNA [42]. The differences in PFAS exposure among the racial divides could be because of differences in lifestyles, geographical locations and/or racial residential segregation, but the differences were independent of the socioeconomic status (SES) of the different races/ethnicity [42].

Similarly, Park and colleagues also studied longitudinal trends in PFAS exposure among multiethnic midlife women in the United States, and the rate of changes in PFAS levels between year 1999 and 2011 was derived using a first-order elimination model [191]. They observed a varied temporal trend in ethnicity/race, with Chinese women having consistently higher concentrations of PFNA than white and black women, while serum PFHxS levels were significantly lowered in white and black women. The findings indicated temporal PFAS exposure trends vary among racial and ethnic groups. Subpopulations with higher initial PFAS exposures tended to experience more significant changes over the study period. Current collection of data of PFAS exposures by ethnicity is proving to be challenging, due to constant changes in the composition of race/ethnicity in the US population because of the higher influx of immigrants, making it extremely difficult to document patterns of human exposure to PFAS [29].

### 3.2. Sociodemographic Variables and Endometrial Cancer

Endometrial cancer (EC), with the aggressive type II subtype that includes high-grade (grade III) endometrioid and non-endometrioid carcinomas, accounts for ~15–20% of all ECs and causes about 45% deaths in all EC cases in the United States [192,193]. The intricate interplay between sociodemographic variables and the development, progression, and outcomes of endometrial cancer are being researched extensively [194,195,196]. The intricate connection also presents a multifaceted landscape that underscores the importance of understanding the role of biology, environment, and society in the continued proliferation of EC in women [197]. Therefore, sociodemographic factors encompass various individual characteristics, including income, education, occupation, zip code or geographical location, and ethnicity, collectively shaping an individual’s social and economic context. These variables, in turn, can influence an individual’s lifestyle choices, access to healthcare, exposure to risk factors, and overall health status.

Data from the National Cancer Institute’s black/white Cancer Survival study, looking at white and black women aged 20–79, was designed to investigate racial differences in survival rates in a population-based sample of patients with cancer. Overall, the 1996 study found that after adjusting for age and zip code/geographical location, black women were four times more likely to die from endometrial cancer than white women. Some of the factors that will be discussed further in this manuscript are presented in Table 2, which summarizes critical sociodemographic factors. The data, though from 1996 [198], still has continued relevance.

#### 3.2.1. Income

In the United States, a nation marked by socioeconomic diversity and advanced healthcare resources, exploring the correlation between endometrial cancer occurrence and high-income households is a crucial endeavor. This review delves into some factors that potentially contribute to the incidence of endometrial cancer among women in affluent households, shedding light on the multifaceted relationship between wealth, health, and disease. Using data from SEER 1988 to 2005, socioeconomic status was calculated based on low (<$20,000/year), intermediate ($20,000–$60,000), and high (>$60,000) income groups [199]. Some researchers are of the opinion that adult populations with higher income have a favorable health outcome when compared to the populations with lower income and they concluded that the lack of access to healthcare for people with lower socioeconomic status is the likely cause [200,201].

In 2004, Madison and co-workers reported that women of higher household incomes rarely present with advanced stage of type II endometrial cancer, while the dynamic shifted negatively for black women from lower household income due to lack of healthcare insurance, limited access to chemotherapy and to hysterectomies [202]. Furthermore, black women of lower socioeconomic status (SES) diagnosed with endometrial cancer exhibited a 2.5-fold increased likelihood of mortality associated with the disease [203,204].

#### 3.2.2. Education

Education frequently correlates with heightened awareness of health risks, improved access to medical resources, and the adoption of healthier behaviors [205]. By concentrating on a demographic distinguished by higher educational attainment, the objective is to reveal potential patterns, risk factors, and preventive measures, which contribute to the comprehension of endometrial cancer epidemiology, encompassing factors such as hormones, lifestyle choices, and environmental influences associated with the risk of endometrial cancer [110,150]. In a recent study conducted among the general gynecologic patient population, the majority of sampled women, regardless of their level of education, demonstrated a lack of awareness regarding the common risks associated with endometrial cancer [206].

Another study found that people living in rural areas with higher poverty and lower educational levels did not present any significant differences in mortality between urban and rural EC patients [207]. In a bid to correlate the cancer with the level of education, other co-morbid factors such as obesity, estrogen use or age were examined, and it was observed that the risk factors for EC did not differ based on women’s level of education [206]. Considering public health and medical research dynamics, a comprehensive exploration of the relationship between educational attainment and endometrial cancer occurrence is imperative.

#### 3.2.3. Occupation

The association of endometrial cancer with the nature of occupation has always been a complex and multifaceted topic. Some studies in the early 2000s suggested that certain occupational exposures may be associated with a higher risk of endometrial cancer [208,209]. For example, exposure to endocrine-disrupting chemicals [210,211] or working in jobs with irregular shift patterns that disrupt circadian rhythms [212,213] could potentially influence the risk. 

The relationship between circadian rhythms disorder and EC onset was comprehensively investigated by Zhang and colleagues [214]. In their study, the sleep patterns and night-shift experiences of endometrial cancer (EC) patients was characterized, and the underlying mechanisms involving the circadian clock gene PER (Period), with a specific focus on the prognostic implications and functional relevance of PER1 expression in relation to night-shift work was investigated. The study involved 619 participants, segregated into two groups based on their engagement in night-shift duties: the rhythm group and the control group. They found a correlation between the onset of EC and night-shift duties, concluding that EC patients have experienced more nightshift and with longer durations [214]. 

However, these associations are often complex and may not apply universally to all individuals in a specific occupation. As a reference, women working in a large hog-concentrated animal feeding operation (CAFO) located in North Carolina, United States, were thought to have an increased odd ratio (ORs) of uterine cancer death, possibly due to the high concentration of endocrine-disrupting chemicals (EDC) around CAFOs [215]. The occupation-related release of heavy metals, such as metalloestrogen cadmium (Cd), and lead (Pb), can cause endometriosis and endometrial cancer [216].

#### 3.2.4. Zip Code or Geographical Location

Zip codes/geographical location can serve as a proxy for various socioeconomic and environmental factors that may influence cancer risk, including access to healthcare, income levels, lifestyle factors, and exposure to environmental toxins. Understanding these associations can help identify areas with higher or lower cancer risk and guide targeted interventions and healthcare resource allocation. According to Barrington et al., Medicaid expansion (ME) was proportional to the number of new EC diagnoses in ME states, and this is usually sorted out using zip codes/geographical location [217,218].

In another study, it was observed that, despite the disparities in income and education, non-Hispanic blacks are more likely to reside in zip codes/geographical locations with the lowest income quartile and are less likely to receive guideline-concordant endometrial cancer treatment [219]. In some daunting situations, patients may sometimes choose to refuse any form of treatment or surgery for endometrial cancer due to various reasons [220,221]. In addition, insurance status and geographical location/zip code-level income were not associated with chemotherapy refusal among the sampled population [217]. It is important to note that while zip codes/geographical location can provide valuable insights into cancer patterns, they are not a direct cause. Instead, they serve as a geographic marker for various risk factors and access to resources that can influence cancer outcomes [222].

#### 3.2.5. Ethnicity

Ethnicity, encompassing cultural, genetic, and social determinants, is another factor that can be used to understand endometrial cancer [223]. Notably, ethnicity often intersects with various factors that directly or indirectly influence cancer risk, such as hormonal profiles, dietary habits, access to healthcare, and genetic predispositions. Consequently, exploring the role of ethnicity in endometrial cancer offers a unique opportunity to dissect the multifaceted nature of this disease. Emerging evidence underscores substantial variations in endometrial cancer incidence and outcomes among different ethnic groups in the United States [224,225].

In a recent edition of the Cancer Statistic publication, Siegel et al. reported an annual increase of 2% (from 2008 to 2018) in the rates of EC occurrence, the cancer with the largest racial disparity in the United States [226]. Siegel and colleagues also observed a changing landscape in EC mortality in the United States, with black women having higher cases of cancer and a mortality rate doubling that of white women (9.1/100,000 vs. 4.6/100,000) [227]. Some of the contributory factors to the widening gap in the burden of endometrial cancer among women of color are not only limited to sociodemographic variables, but also encompass clinicopathologic and treatment factors [192,228].

Understanding the role of ethnicity in endometrial cancer is crucial for developing targeted prevention strategies, improving healthcare access, and addressing health disparities. The authors believed that genetic differences among ethnicities can indeed play a significant role in the risk, development, and outcomes of endometrial cancer in the United States. These genetic distinctions can influence susceptibility, tumor characteristics, and responses to treatments [229].

### 3.3. Psychosocial and Environmental Stress on Endometrial Cancer

A possible mediation between PFAS exposure and EC risk is possible through various pathways involving chronic stress responses, hormonal dysregulation, inflammation, oxidative stress, immune dysfunction, and behavioral factors. Understanding these interactions is essential for elucidating the etiology of EC and formulating holistic approaches for prevention and intervention. PFAS is a potent endocrine disruptor, and various studies allude to its devastating effect in interacting with hormone receptors and pathways in the body, potentially leading to hormonal dysregulation [230,231]. A recent study found a strong correlation between several PFAS and some hormones such as DHEAS, thyroid, parathyroid, and thyroid hormones in both girls and boys [232]. Yi et al. found an association of PFAS serum concentrations with depression [233], a known psychosocial stress that has been implicated in EC risk [234]. Another study examined the effect of PFAS and psychosocial stress on birthweight for gestational age among African Americans in the Atlanta region. The researchers concluded that PFAS and psychosocial stressors positively correlated with lower birthweight for gestational age z-scores, but these correlations were strongest when PFAS and the stressors were modeled as a mixture [235].

## 4. Discussion

The relationship between sociodemographic variables and endometrial cancer, as well as the relationship between sociodemographic factors and PFAS, reveals a significant overlap in their impact. Factors such as ethnicity, zip code or geographical location, occupation, education, and income play pivotal roles in both realms. Ethnicity often intertwines with geographical locations (zip codes), influencing exposure levels to environmental factors, including PFAS, and potentially impacting endometrial cancer rates. Moreover, occupation and income levels can directly influence exposure to certain environmental contaminants like PFAS, consequently impacting health outcomes, including potential risks for endometrial cancer. Education is pivotal in understanding and mitigating risks associated with environmental exposure and health outcomes, highlighting the intersection between these variables. Educational level may impact awareness and understanding of environmental risks and health practices. Occupation often determines the degree of direct or indirect exposure to PFAS, particularly in industries using these substances. The zip code or geographical location is significant, as it can reflect the environmental pollution levels in an area and the proximity to PFAS sources. Lastly, ethnicity may be linked to varying levels of exposure and different genetic susceptibilities to the effects of PFAS, further influencing the risk of endometrial cancer. These sociodemographic variables serve as crucial indicators, showcasing the complex relationship between environmental factors like PFAS, sociodemographic elements, and the prevalence of endometrial cancer. However, due to the limitations in the current literature, there is a need for more specific studies that can isolate and analyze the impact of these sociodemographic factors uniquely on endometrial cancer, especially in the context of PFAS exposure.

### 4.1. PFAS and Its Effects on Endometrial Cancer

The relationship between PFAS (per- and polyfluoroalkyl substances) and endometrial cancer is an emerging area of research. While evidence is still evolving, several mechanisms have been proposed to explain the potential link between PFAS exposure and the development of endometrial cancer. PFAS have been implicated as endocrine disruptors by interfering with the action of hormones (such as estrogen), and this could potentially lead to endometrial cancer in females [2,3]. However, this claim has not been substantially proven by scientists.

In a study published in 2016 by Ma and colleagues, PFOA was found to induce human Ishikawa endometrial cancer cell invasion and migration. They also found that the mechanism of activation was through induction of ERK1/2/mTOR signaling [236].

### 4.2. Toxicological Mechanisms

#### 4.2.1. Endocrine Disruption

PFAS compounds are well-documented for their endocrine-disrupting properties, with notable links to endometrial cancer (EC) [210,237]. Numerous studies have revealed estrogenic and anti-androgenic activities associated with various PFAS compounds. For instance, PFOA has been linked to uterine changes and reproductive health issues in female mice [2,238,239]. While it is widely acknowledged that endocrine-disrupting chemicals (EDCs), including PFAS, can interact with nuclear receptors, such as peroxisome proliferator-activated receptors (PPARs), the direct activation of specific endocrine receptors like estrogen (ER) and androgen receptors (AR) by PFAS remains less established [20,240]. As a result, the precise mechanism underlying PFAS-mediated endocrine disruption remains elusive. This suggests that indirect pathways, possibly involving epigenetic modifications or metabolic reprogramming, might contribute to the disruption of endocrine hormone production and secretion during critical exposure periods.

#### 4.2.2. Epigenetics

Despite the growing recognition of non-mutagenic epigenetic pathways as pivotal in the biological effects of PFAS, research in this domain has been relatively limited, primarily focusing on DNA methylation [241,242,243]. PFAS exposure has been associated with both hypomethylation and hypermethylation in genome-wide and gene-specific molecular epidemiology studies. To date, however, mechanistic studies investigating these epigenetic effects remain sparse. It is plausible that PFAS-induced epigenetic reprogramming may be influenced, at least in part, by alterations in metabolites and cofactors that affect epigenetic enzyme activity [244,245]. This epigenetic-mediated transcriptional reprogramming may play a crucial role in establishing and sustaining the metabolic and hormonal conditions necessary for ongoing tumorigenesis, even though the precise mechanisms require further exploration and elucidation.

#### 4.2.3. Epidemiology Studies

Within the domain of epidemiological research, the current body of literature has primarily centered its attention on perfluorooctanoic acid (PFOA) in the context of its potential link to human cancer [236,246,247,248]. This investigation unfolds across three distinct population cohorts, each contributing unique insights to our understanding of the relationship between PFAS exposure and cancer risk.

Firstly, the occupational exposure cohort delves into individuals who have come into contact with PFAS, particularly PFOA, within the scope of their employment. These individuals work in facilities that either produce or employ PFAS compounds, making them a focal point for assessing the potential health hazards associated with direct PFAS exposure in occupational settings. Considering the relationships between two risk factors, it was noted that previous occupational exposure studies confirmed that there is no direct correlation between PFAS (e.g., PFOA/PFOS) and uterine cancer [249]. A recent study on occupational mortality, which investigated the link between cancer rates and exposure to PFAS, found no significant association between the two factors in terms of health outcomes. [250,251]. However, these studies selected a limited sample size and a limitation to sample areas also occurred, with a wide confidence interval (1.–4.98) [252].

Secondly, the community exposure cohort comprises residents of areas where documented contamination of the local environment or drinking water supply with PFAS, including PFOA, has been identified. Research within this cohort seeks to illuminate the health implications and potential cancer-related risks associated with residing in regions affected by PFAS contamination, offering insights into community-wide health consequences. A recent study by a group of researchers found out that environmental PFAS exposure has stronger connections with carcinogenicity in rats, and the exposure also resulted in an elevated risk of cancer [16,253]. In retrospect, a study revealed environmental exposure to PFOS and PFOA increased the risk of cancer diagnosis, with a 3.3-fold increase in cancer mortality in the PFOA industry [254]. However, upon reanalysis of the cohort, it was found that there was no link between exposure and cancer incidence [255], and this report was consistent with previous studies [250,251].

Lastly, the background exposure cohort encompasses the general population, encompassing individuals whose PFAS exposure levels align with typical or background concentrations. This segment of the population serves as a lens through which researchers can evaluate the potential cancer-related implications of PFAS exposure, even at lower and more prevalent exposure levels within the broader populace. A study investigated the impact of background concentrations of PFAS on African American and non-Hispanic white women, and it was concluded that exposure to PFAS was directly related to the behavioral patterns of mostly African American women [190]. Frequent consumption of prepared food in coated cardboard containers, flossing with Oral-B Glide, having stain-resistant carpet, or living around water bodies contaminated with PFAS are disproportionately towards African American women [190,256]. Epidemiological studies conducted by Imir et al. also confirmed the link between increased blood PFAS levels and prostate cancer incidence [256]. In the case of endometriosis, 1.3 per 1000 women aged 15–49 sampled in a general population have either confirmed endometrial cancer surgically or pathologically, according to a 2016 study [257]. Epidemiological findings have validated the prevalence of EC between 1 and 10% by various methods of diagnosis, lifestyle factors, and fertility status [38].

### 4.3. Stress Relationships

#### 4.3.1. PFAS

PFAS, like many environmental pollutants, has been associated with stress [5,6]. Due to the health burden caused by PFAS exposure, stress has been known to affect the body’s hormonal balance, immune function, and inflammation levels, all of which could potentially play a role in cancer development. For example, research has found a relationship between PFAS exposure and allostatic load, an index of chronic stress [9,10,11]. This indicates that the biological response to stress increases when exposed to PFAS. In a recent investigation involving 3193 participants from the pre- and post-menopausal population, serum PFAS levels were examined alongside biological aging processes using data from NHANES 2003 to 2018. The study revealed a variable influence of PFAS exposure on biological aging, highlighting a dependence on specific physiological processes [258]. In addition, its relationship with sociodemographic and economic variables likely intersects with this finding of a relationship.

Eick and colleagues investigated the association of PFAS and maternal stress and its effect on birth outcomes and offspring neurodevelopment [162]. The study considered associations of PFAS exposures and psychosocial stress with demographic characteristics using linear models. They found out that median levels of PFOS are higher in the infants delivered by women with higher levels of psychosocial stress level. They also concluded that demographic characteristics did not contribute to the cognitive outcomes of the infants [162].

A recent study also found lower birthweight-for-gestational-age z-scores for infants born by women affected by PFAS and psychosocial stress during pregnancy [259]. Research continues to abound on the positive correlation of PFAS and stress [235,260,261,262], and in another study, using data (*n* = 6652) from NHANES 2005 to 2012, the researchers found that serum bilirubin, albumin, and iron, critical indicators of oxidative stress, were continuously increasing in participants exposed to PFAS [263].

#### 4.3.2. Endometrial Cancer

The relationship between endometrial cancer and stress is a complex one, and it is important to note that no single factor, including stress, can definitively cause cancer [139]. In a study carried out in 2007 by Nielsen and colleagues, a weak correlation between stress and EC was found in the severely stressed group; in fact, a lower risk of EC was observed among stressed women [264]. Due to the small, sampled population (*n* = 72), several research studies have focused on a much larger and diverse sampling method [265,266], and a study of 250 patients and multiple races/ethnicities found out that stress played a key role in the development of EC [265].

In a study evaluating the quality of life and emotional distress in endometrial patients, Ferrandina and colleagues found diminished quality of life and increased distress among EC patients in a 2014 study [267]. Reid and colleagues likewise found distress levels to be high among women with EC, with race playing a role in distress levels [268]. Oxidative stress (OS) was also implicated in the development of EC, as the markers for serum OS were elevated in all patients with EC [269].

#### 4.3.3. PFAS Contributing to Endometrial Cancer

Emerging research suggests a compelling connection between PFAS (per- and polyfluoroalkyl substances) exposure and stress indices such as allostatic load, which in turn may contribute to the development of chronic diseases, including endometrial cancer. PFAS compounds, notorious for their persistence in the environment and bioaccumulation within living organisms, have been linked to endocrine disruption and immune system dysregulation [86,270]. These disruptive effects can elevate allostatic load, a measure of cumulative stress on the body, as the body’s stress response systems continually react to the presence of these pollutants. Elevated allostatic load is recognized as a mediator in developing various chronic diseases, including cancer [271,272]. In the specific context of endometrial cancer, the potential for PFAS-induced stress to contribute to carcinogenesis warrants thorough investigation. Understanding the intricate relationship between PFAS, allostatic load, and endometrial cancer may provide crucial insights into the mechanistic pathways underpinning this disease and inform preventive measures and interventions for at-risk populations.

PFAS compounds are known environmental stressors. They can disrupt the endocrine system, induce oxidative stress, and lead to immune system dysregulation. These stress-inducing effects are pivotal in elevating allostatic load. Allostatic load measures the cumulative physiological toll of chronic stress responses, including hormonal changes, immune responses, and oxidative damage. As the body continually reacts to the presence of PFAS, this load increases over time [86]. In endometrial cancer, an elevated allostatic load may play a pivotal role in developing and progressing cancerous cells within the endometrium [7,8]. The mechanisms through which allostatic load contributes to endometrial cancer development involve several interconnected factors, such as hormonal imbalances, immune system dysregulation, and oxidative stress.

PFAS-induced stress can lead to hormonal imbalances, including disruptions in estrogen regulation. These imbalances can promote the growth of cancerous cells in the endometrium. As estrogen plays a critical role in endometrial tissue regulation, any disturbance in its balance is a significant risk factor [273,274]. PFAS exposure can also interfere with immune system function, influencing the body’s ability to detect and eliminate cancer cells. A compromised immune system may be less effective at preventing the development and progression of endometrial cancer [275,276]. In addition, PFAS exposure has been associated with oxidative stress, which can lead to DNA damage. This DNA damage is a known factor in the development of cancer, including endometrial cancer. Oxidative stress induced by PFAS exposure can create an environment conducive to cancer initiation and progression [277,278].

### 4.4. Limitations

The review investigating the association of PFAS and endometrial cancer encountered several limitations. Primarily, the reliance on existing literature and available data for PFAS exposure and endometrial cancer risk posed a challenge due to variations in study methodologies. Heterogeneity among studies in methodologies, exposure assessment, and outcome measurements hindered drawing definitive conclusions. Moreover, establishing causality between PFAS exposure and endometrial cancer risk proved complex due to confounding factors such as lifestyle, genetics, and other environmental exposures. Additionally, limitations in generalizability to diverse populations, temporal factors, absence of longitudinal studies, potential exposure misclassification, and the evolving nature of research in this area were noteworthy constraints encountered in the review process. Acknowledging these limitations is essential for a nuanced interpretation of the review’s findings and for guiding future research endeavors in understanding the relationship between PFAS exposure and endometrial cancer risk, and the role of exposure sources, sociodemographic factors, and stressors.

Overall, this preliminary assessment of the relationship between the study’s factors is critical [279,280] and provides a key insight into how social factors may explain PFAS exposure risk and EC outcomes.

## 5. Conclusions

Per- and polyfluoroalkyl substances (PFAS), due to their unique chemical properties, currently have high application in the industry for producing broad range of consumer products. Pollution from PFAS is on the increase, and its stability, due to the C–F bond’s strength, is a major environmental concern. PFAS is persistent and recalcitrant in nature and is therefore commonly found in water (both drinking and underground water), air, and soil. Humans can be exposed to PFAS by consuming contaminated water, animal, or consumer products. Exposure can also be dependent on some sociodemographic indices. Occupation poses a significant exposure risk for humans, especially concerning PFAS-containing fire-fighting foams, widely utilized by firefighting departments. Prolonged exposure to PFAS presents a considerable health hazard, as it is associated with thyroid dysfunction, different cancers, diabetes, high cholesterol, elevated liver enzymes, and various other disorders [86].

The paper focused on how PFAS exposure may lead EC. Despite technological advancements in early detection and treatment, endometrial cancer (EC) continues to impose a higher health burden than most cancers affecting women, underscoring the urgency healthcare solutions and prevention strategies. The epidemiology of EC provided valuable insights into its pervasiveness, likelihood, and impact on public health. Disparities in EC cases among different racial groups in the United States were found to be dependent on socioeconomic status (SES), biological differences, and healthcare access, highlighting the need for healthcare policies that address health equity. PFAS is thought to interact with female hormones through the disruption of endocrine glands, presenting a concern related to hormonal balance and reproductive health. This toxicological mechanism of action could potentially lead to EC, emphasizing the need for chemical management practices and regulation. One possible cause of PFAS-induced EC could be epigenetic reprogramming, leading to alterations in metabolites and cofactors responsible for epigenetic enzyme activity.

In this study we found that PFAS exposure can lead to oxidative stress, thus creating a platform for cancer initiation and progression, emphasizing the importance of environmental protection measures to reduce oxidative stressors in ecosystems. It is noteworthy that the review focused on an exploratory association of PFAS and the risk of EC. Future studies should prioritize achieving an accurate prognosis for PFAS-induced EC. Future research should prioritize longitudinal studies examining cumulative PFAS exposure’s relationship with endometrial cancer risk, elucidating underlying mechanisms like hormonal disruption and oxidative stress. Identifying specific biomarkers and conducting population-based analyses across diverse demographics will aid in early detection and risk assessment, informing effective intervention strategies and policy decisions to reduce PFAS-related health hazards.

## Figures and Tables

**Table 1:** Influence of sociodemographic data on the concentrations of PFAS.

	*n* (%)	PFOA Median (µg/L)	PFOS Median (µg/L)	PFNA Median (µg/L)	PFHxS Median (µg/L)
**NHANES cycle**					
2003–2004	1929 (49)	3.8	19.8	0.9	1.9
2005–2006	2024 (51)	3.7	16.0	1.0	1.7
**Age**					
12–19	1196 (30)	3.7	16.0	0.9	2.1
20–59	1795 (45)	3.7	17.0	1.0	1.6
≥60	962 (24)	4.0	23.5	1.0	1.9
**Family income**					
$0–$19,999	1185 (30)	3.4	16.5	0.9	1.7
$20,000–$44,900	1326 (34)	3.7	17.9	0.9	1.8
$45,000–$74,999	735 (19)	4.0	18.5	1.0	1.8
≥$75,000	707 (18)	4.2	19.8	1.1	2.0
**Education**					
<High school	780 (28)	3.4	18.3	0.9	1.7
High school/GED	686 (25)	4.0	19.1	1.0	1.7
Associate degree	782 (28)	3.9	18.9	0.9	1.7
College grad and above	508 (18)	4.1	21.0	1.1	1.8
**Occupation type**					
Never worked	142 (9)	3.5	17.4	0.7	2.1
Blue collar, semi-routine	684 (42)	3.9	19.6	0.9	1.9
Blue collar, high skill	226 (13)	3.6	22.0	0.9	1.7
white collar, semi-routine	289 (18)	3.8	19.6	0.9	1.7
white collar and professional	311 (19)	3.9	21.8	1.0	1.8
**Race/Ethnicity**					
Non-Hispanic white	1781 (45)	4.2	19.9	1.0	1.9
Non-Hispanic black	1013 (26)	3.7	19.5	1.0	1.9
* Hispanic-Mexican	469 (12)	3.5	15.4	0.7	1.7
Other Hispanic	117 (3)	3.8	14.8	1.0	1.7
Other race/multiracial	142 (4)	3.6	18.6	1.0	2.0

Education includes only those over 20, and occupation is age 16 and above. * Only includes Mexican born in the United States. Table adapted from Nelson et al. [154].

**Table 2:** Sociodemographic factors stratified by race for women with endometrial cancer.

	Black		White		
	*n*	%	*n*	%	*p*-Value
Poverty Index *					
≤100	36	27.7	23	7.0	<0.001
101–220	24	18.5	43	13.1	<0.001
>200	18	13.8	157	47.7	<0.001
Unknown	52	40	106	32.2	<0.001
**Education**					
0–8	34	26.2	20	6.1	<0.001
9–11	17	13.1	29	8.8	<0.001
12	23	17.7	88	26.7	<0.001
>12 Unknown	18 38	13.8 29.2	117 75	35.6 22.8	<0.001
**Occupation type**					
Managerial/Professional	9	6.9	54	16.4	<0.001
Homemaker	11	8.5	50	15.2	<0.001
Technical/Sales/Admin	10	7.7	96	29.2	<0.001
Skilled Laborer	35	26.9	36	10.9	<0.001
Unskilled Laborer	26	20	16	4.9	<0.001
--- **Stage**		-			
I II III IV	85 13 17 16	65 10 13.1 11.5	276 22 21 10	83.9 6.7 6.4 3.0	<0.001 <0.001 <0.001 <0.001
**Pathologic Grade**	45	34.9	173	52.7	<0.001
Well differentiated Moderately differentiated Poorly differentiated Unknown	46 38 1	35.7 29.5	106 49 1	32.3 14.9	<0.001 <0.001

* Poverty index obtained by dividing household income by US poverty level income for a family of the corresponding size. Table adapted from Hill et al. [198].

## References

[1] Zhu Y., Ro A., Bartell S.M. (2021). Household low pile carpet usage was associated with increased serum PFAS concentrations in 2005–2006. Environ. Res..

[2] Rickard B.P., Rizvi I., Fenton S.E. (2022). Fenton, Per- and poly-fluoroalkyl substances (PFAS) and female reproductive outcomes: PFAS elimination, endocrine-mediated effects, and disease. Toxicology.

[3] Ding N., Harlow S.D., Randolph J.F., Loch-Caruso R., Park S.K. (2020). Perfluoroalkyl and polyfluoroalkyl substances (PFAS) and their effects on the ovary. Hum. Reprod. Update.

[4] Ao J., Zhang R., Huo X., Zhu W., Zhang J. (2024). Environmental exposure to legacy and emerging per- and polyfluoroalkyl substances and endometriosis in women of childbearing age. Sci. Total Environ..

[5] Eick S.M., Enright E.A., Padula A.M., Aung M., Geiger S.D., Cushing L., Trowbridge J., Keil A.P., Gee Baek H., Smith S. (2022). Prenatal PFAS and psychosocial stress exposures in relation to fetal growth in two pregnancy cohorts: Applying environmental mixture methods to chemical and non-chemical stressors. Environ. Int..

[6] Taibl K.R., Schantz S., Aung M.T., Padula A., Geiger S., Smith S., Park J.S., Milne G.L., Robinson J.F., Woodruff T.J. (2022). Associations of per- and polyfluoroalkyl substances (PFAS) and their mixture with oxidative stress biomarkers during pregnancy. Environ. Int..

[7] McEwen B.S. (1998). Protective and damaging effects of stress mediators. N. Engl. J. Med..

[8] Hollar D.W. (2022). Allostatic load, mobility disability, and viral effects in cancer: A structural equation model. Cancer Investig..

[9] Bashir T., Obeng-Gyasi E. (2022). The Association between Multiple Per-and Polyfluoroalkyl Substances’ Serum Levels and Allostatic Load. Int. J. Environ. Res. Public Health.

[10] Bashir T., Obeng-Gyasi E. (2023). Combined Effects of Multiple Per-and Polyfluoroalkyl Substances Exposure on Allostatic Load Using Bayesian Kernel Machine Regression. Int. J. Environ. Res. Public Health.

[11] Boafo Y., Mostafa S., Obeng-Gyasi E. (2023). Association of Per-and Polyfluoroalkyl Substances with Allostatic Load Stratified by Herpes Simplex Virus 1 and 2 Exposure. Toxics.

[12] Messmer M.F., Salloway J., Shara N., Locwin B., Harvey M.W., Traviss N. (2022). Risk of cancer in a community exposed to per-and poly-fluoroalkyl substances. Environ. Health Insights.

[13] Bartell S.M., Vieira V.M. (2021). Critical review on PFOA, kidney cancer, and testicular cancer. J. Air Waste Manag. Assoc..

[14] Rickard B.P., Overchuk M., Tulino J., Tan X., Ligler F.S., Bae-Jump V.L., Fenton S.E., Rizvi I. (2023). Exposure to select PFAS and PFAS mixtures alters response to platinum-based chemotherapy in endometrial cancer cell lines. Environ. Health.

[15] Ehrlich V., Bil W., Vandebriel R., Granum B., Luijten M., Lindeman B., Grandjean P., Kaiser A.-M., Hauzenberger I., Hartmann C. (2023). Consideration of pathways for immunotoxicity of per-and polyfluoroalkyl substances (PFAS). Environ. Health.

[16] Temkin A.M., Hocevar B.A., Andrews D.Q., Naidenko O.V., Kamendulis L.M. (2020). Application of the Key Characteristics of Carcinogens to Per and Polyfluoroalkyl Substances. Int. J. Environ. Res. Public Health.

[17] Kjeldsen L.S., Bonefeld-Jørgensen E.C. (2013). Perfluorinated compounds affect the function of sex hormone receptors. Environ. Sci. Pollut. Res..

[18] Ansari R.A., Alfuraih S., Shekh K., Omidi Y., Javed S., Shakil S.A. (2023). Endocrine Disruptors: Genetic, Epigenetic, and Related Pathways. Impact of Engineered Nanomaterials in Genomics and Epigenomics.

[19] Bapat S.A. (2012). Epigenetic regulation of cancer stem cell gene expression. Epigenetics: Development and Disease.

[20] Kim S., Thapar I., Brooks B.W. (2021). Epigenetic changes by per-and polyfluoroalkyl substances (PFAS). Environ. Pollut..

[21] Chen H., Wei S., Li J., Zhong Z., Chen D. (2023). Transplacental transport of Per-and polyfluoroalkyl substances (PFAS): Mechanism exploration via BeWo cell monolayer model. J. Hazard. Mater..

[22] Al Amin M., Sobhani Z., Liu Y., Dharmaraja R., Chadalavada S., Naidu R., Chalker J.M., Fang C. (2020). Recent advances in the analysis of per- and polyfluoroalkyl substances (PFAS)—A review. Environ. Technol. Innov..

[23] Boiteux V., Dauchy X., Rosin C., Munoz J.F. (2012). National screening study on 10 perfluorinated compounds in raw and treated tap water in France. Arch. Environ. Contam. Toxicol..

[24] Christensen E.R., Wang Y., Huo J., Li A. (2022). Properties and fate and transport of persistent and mobile polar organic water pollutants: A review. J. Environ. Chem. Eng..

[25] Li Y., Andersson A., Xu Y., Pineda D., Nilsson C.A., Lindh C.H., Jakobsson K., Fletcher T. (2022). Determinants of serum half-lives for linear and branched perfluoroalkyl substances after long-term high exposure—A study in Ronneby, Sweden. Environ. Int..

[26] Xu Y., Fletcher T., Pineda D., Lindh C.H., Nilsson C., Glynn A., Vogs C., Norstrom K., Lilja K., Jakobsson K. (2020). Serum Half-Lives for Short- and Long-Chain Perfluoroalkyl Acids after Ceasing Exposure from Drinking Water Contaminated by Firefighting Foam. Environ. Health Perspect..

[27] Selahle S.K., Mpupa A., Nomngongo P.N. (2022). Liquid chromatographic determination of per- and polyfluoroalkyl substances in environmental river water samples. Arab. J. Chem..

[28] Li Y., Fletcher T., Mucs D., Scott K., Lindh C.H., Tallving P., Jakobsson K. (2018). Half-lives of PFOS, PFHxS and PFOA after end of exposure to contaminated drinking water. Occup. Environ. Med..

[29] Jain R.B. (2018). Time trends over 2003–2014 in the concentrations of selected perfluoroalkyl substances among US adults aged >/=20 years: Interpretational issues. Sci Total Environ..

[30] Blake B.E., Fenton S.E. (2020). Early life exposure to per- and polyfluoroalkyl substances (PFAS) and latent health outcomes: A review including the placenta as a target tissue and possible driver of peri- and postnatal effects. Toxicology.

[31] Brennan N.M., Evans A.T., Fritz M.K., Peak S.A., von Holst H.E. (2021). Trends in the Regulation of Per- and Polyfluoroalkyl Substances (PFAS): A Scoping Review. Int. J. Environ. Res. Public Health.

[32] He Y., Cheng X., Gunjal S.J., Zhang C. (2023). Advancing PFAS Sorbent Design: Mechanisms, Challenges, and Perspectives. ACS Materials Au.

[33] Yadav M., Osonga F.J., Sadik O.A. (2024). Unveiling nano-empowered catalytic mechanisms for PFAS sensing, removal and destruction in water. Sci. Total Environ..

[34] Marquínez-Marquínez A.N., Loor-Molina N.S., Quiroz-Fernández L.S., Maddela N.R., Luque R., Rodríguez-Díaz J.M. (2023). Recent advances in the remediation of perfluoroalkylated and polyfluoroalkylated contaminated sites. Environ. Res..

[35] Sinclair G.M., Long S.M., Jones O.A. (2020). What are the effects of PFAS exposure at environmentally relevant concentrations?. Chemosphere.

[36] Garg A., Shetti N.P., Basu S., Nadagouda M.N., Aminabhavi T.M. (2023). Treatment technologies for removal of per- and polyfluoroalkyl substances (PFAS) in biosolids. Chem. Eng. J..

[37] Dadashi Firouzjaei M., Zolghadr E., Ahmadalipour S., Taghvaei N., Akbari Afkhami F., Nejati S., Elliott M.A. (2021). Chemistry, abundance, detection and treatment of per- and polyfluoroalkyl substances in water: A review. Environ. Chem. Lett..

[38] Andrews D.Q., Hayes J., Stoiber T., Brewer B., Campbell C., Naidenko O.V. (2021). Identification of point source dischargers of per- and polyfluoroalkyl substances in the United States. AWWA Water Sci..

[39] Solo-Gabriele H.M., Jones A.S., Lindstrom A.B., Lang J.R. (2020). Waste type, incineration, and aeration are associated with per- and polyfluoroalkyl levels in landfill leachates. Waste Manag..

[40] Ng K., Alygizakis N., Androulakakis A., Galani A., Aalizadeh R., Thomaidis N.S., Slobodnik J. (2022). Target and suspect screening of 4777 per- and polyfluoroalkyl substances (PFAS) in river water, wastewater, groundwater and biota samples in the Danube River Basin. J. Hazard. Mater..

[41] DeLuca N.M., Thomas K., Mullikin A., Slover R., Stanek L.W., Pilant A.N., Cohen Hubal E.A. (2023). Geographic and demographic variability in serum PFAS concentrations for pregnant women in the United States. J. Expo. Sci. Environ. Epidemiol..

[42] Park S.K., Peng Q., Ding N., Mukherjee B., Harlow S.D. (2019). Determinants of per- and polyfluoroalkyl substances (PFAS) in midlife women: Evidence of racial/ethnic and geographic differences in PFAS exposure. Environ. Res..

[43] Ba S.A., Meng Q. (2021). PFASs in Consumer Products: Exposures and Regulatory Approaches. Forever Chemicals.

[44] Rodgers K.M., Swartz C.H., Occhialini J., Bassignani P., McCurdy M., Schaider L.A. (2022). How Well Do Product Labels Indicate the Presence of PFAS in Consumer Items Used by Children and Adolescents?. Environ. Sci. Technol..

[45] Das S., Ronen A. (2022). A Review on Removal and Destruction of Per- and Polyfluoroalkyl Substances (PFAS) by Novel Membranes. Membranes.

[46] Glüge J., Scheringer M., Cousins I.T., DeWitt J.C., Goldenman G., Herzke D., Lohmann R., Ng C.A., Trier X., Wang Z. (2020). An overview of the uses of per-and polyfluoroalkyl substances (PFAS). Environ. Sci. Process. Impacts.

[47] Kotthoff M., Muller J., Jurling H., Schlummer M., Fiedler D. (2015). Perfluoroalkyl and polyfluoroalkyl substances in consumer products. Environ. Sci. Pollut. Res. Int..

[48] Xia C., Diamond M.L., Peaslee G.F., Peng H., Blum A., Wang Z., Shalin A., Whitehead H.D., Green M., Schwartz-Narbonne H. (2022). Per- and Polyfluoroalkyl Substances in North American School Uniforms. Environ. Sci. Technol..

[49] Beesoon S., Genuis S.J., Benskin J.P., Martin J.W. (2012). Exceptionally high serum concentrations of perfluorohexanesulfonate in a Canadian family are linked to home carpet treatment applications. Environ. Sci. Technol..

[50] Chen J., Tang L., Chen W.-Q., Peaslee G.F., Jiang D. (2020). Flows, stock, and emissions of poly-and perfluoroalkyl substances in California carpet in 2000–2030 under different scenarios. Environ. Sci. Technol..

[51] Wu Y., Romanak K., Bruton T., Blum A., Venier M. (2020). Per- and polyfluoroalkyl substances in paired dust and carpets from childcare centers. Chemosphere.

[52] Graber J.M., Black T.M., Shah N.N., Caban-Martinez A.J., Lu S.E., Brancard T., Yu C.H., Turyk M.E., Black K., Steinberg M.B. (2021). Prevalence and Predictors of Per- and Polyfluoroalkyl Substances (PFAS) Serum Levels among Members of a Suburban US Volunteer Fire Department. Int. J. Environ. Res. Public Health.

[53] Harris M.H., Rifas-Shiman S.L., Calafat A.M., Ye X., Mora A.M., Webster T.F., Oken E., Sagiv S.K. (2017). Predictors of Per- and Polyfluoroalkyl Substance (PFAS) Plasma Concentrations in 6–10 Year Old American Children. Environ. Sci. Technol..

[54] Sunderland E.M., Hu X.C., Dassuncao C., Tokranov A.K., Wagner C.C., Allen J.G. (2019). A review of the pathways of human exposure to poly- and perfluoroalkyl substances (PFASs) and present understanding of health effects. J. Expo. Sci. Environ. Epidemiol..

[55] Post G.B. (2021). Recent US State and Federal Drinking Water Guidelines for Per- and Polyfluoroalkyl Substances. Environ. Toxicol. Chem..

[56] Stoiber T., Evans S., Temkin A.M., Andrews D.Q., Naidenko O.V. (2020). PFAS in drinking water: An emergent water quality threat. Water Solut..

[57] United States Environmental Protection Agency (2023). Proposed PFAS National Primary Drinking Water Regulation. https://www.epa.gov/sdwa/and-polyfluoroalkyl-substances-pfas.

[58] Babayev M., Capozzi S.L., Miller P., McLaughlin K.R., Medina S.S., Byrne S., Zheng G., Salamova A. (2022). PFAS in drinking water and serum of the people of a southeast Alaska community: A pilot study. Environ. Pollut..

[59] Andrews D.Q., Naidenko O.V. (2020). Population-Wide Exposure to Per- and Polyfluoroalkyl Substances from Drinking Water in the United States. Environ. Sci. Technol. Lett..

[60] Voulgaropoulos A. (2022). Mitigation of PFAS in U.S. Public Water Systems: Future steps for ensuring safer drinking water. Environ. Prog. Sustain. Energy.

[61] Sungur Ş., Çevik B., Köroğlu M. (2022). Determination of perfluorooctanoic acid (PFOA) and perfluorooctane sulfonic acid (PFOS) contents of compost amended soils and plants grown in these soils. Int. J. Environ. Anal. Chem..

[62] Domingo J.L., Nadal M. (2019). Human exposure to per- and polyfluoroalkyl substances (PFAS) through drinking water: A review of the recent scientific literature. Environ. Res..

[63] Li Y., Liu Y., Shi G., Liu C., Hao Q., Wu L. (2022). Occurrence and Risk Assessment of Perfluorooctanoate (PFOA) and Perfluorooctane Sulfonate (PFOS) in Surface Water, Groundwater and Sediments of the Jin River Basin, Southeastern China. Bull. Environ. Contam. Toxicol..

[64] Kotlarz N., McCord J., Collier D., Lea C.S., Strynar M., Lindstrom A.B., Wilkie A.A., Islam J.Y., Matney K., Tarte P. (2020). Measurement of Novel, Drinking Water-Associated PFAS in Blood from Adults and Children in Wilmington, North Carolina. Environ. Health Perspect..

[65] Boone J.S., Vigo C., Boone T., Byrne C., Ferrario J., Benson R., Donohue J., Simmons J.E., Kolpin D.W., Furlong E.T. (2019). Per- and polyfluoroalkyl substances in source and treated drinking waters of the United States. Sci. Total Environ..

[66] Zhu Y., Bartell S.M. (2022). Per- and polyfluoroalkyl substances in drinking water and hypertensive disorders of pregnancy in the United States during 2013–2015. Environ. Epidemiol..

[67] Petre M.A., Genereux D.P., Koropeckyj-Cox L., Knappe D.R.U., Duboscq S., Gilmore T.E., Hopkins Z.R. (2021). Per- and Polyfluoroalkyl Substance (PFAS) Transport from Groundwater to Streams near a PFAS Manufacturing Facility in North Carolina, USA. Environ. Sci. Technol..

[68] Pritchett J.R., Rinsky J.L., Dittman B., Christensen A., Langley R., Moore Z., Fleischauer A.T., Koehler K., Calafat A.M., Rogers R. (2019). Notes from the Field: Targeted Biomonitoring for GenX and Other Per- and Polyfluoroalkyl Substances Following Detection of Drinking Water Contamination—North Carolina, 2018. MMWR Morb. Mortal. Wkly. Rep..

[69] Fan X., Tang S., Wang Y., Fan W., Ben Y., Naidu R., Dong Z. (2022). Global Exposure to Per- and Polyfluoroalkyl Substances and Associated Burden of Low Birthweight. Environ. Sci. Technol..

[70] Gyllenhammar I., Diderholm B., Gustafsson J., Berger U., Ridefelt P., Benskin J.P., Lignell S., Lampa E., Glynn A. (2018). Perfluoroalkyl acid levels in first-time mothers in relation to offspring weight gain and growth. Environ. Int..

[71] Wikstrom S., Lin P.I., Lindh C.H., Shu H., Bornehag C.G. (2020). Maternal serum levels of perfluoroalkyl substances in early pregnancy and offspring birth weight. Pediatr. Res..

[72] Johnson P.I., Sutton P., Atchley D.S., Koustas E., Lam J., Sen S., Robinson K.A., Axelrad D.A., Woodruff T.J. (2014). The Navigation Guide—Evidence-based medicine meets environmental health: Systematic review of human evidence for PFOA effects on fetal growth. Environ. Health Perspect..

[73] Cordner A., De La Rosa V.Y., Schaider L.A., Rudel R.A., Richter L., Brown P. (2019). Guideline levels for PFOA and PFOS in drinking water: The role of scientific uncertainty, risk assessment decisions, and social factors. J. Expo. Sci. Environ. Epidemiol..

[74] Grandjean P. (2018). Delayed discovery, dissemination, and decisions on intervention in environmental health: A case study on immunotoxicity of perfluorinated alkylate substances. Environ. Health.

[75] Bolan N., Sarkar B., Yan Y., Li Q., Wijesekara H., Kannan K., Tsang D.C.W., Schauerte M., Bosch J., Noll H. (2021). Remediation of poly- and perfluoroalkyl substances (PFAS) contaminated soils—To mobilize or to immobilize or to degrade?. J. Hazard. Mater..

[76] Roth J., Abusallout I., Hill T., Holton C., Thapa U., Hanigan D. (2020). Release of Volatile Per- and Polyfluoroalkyl Substances from Aqueous Film-Forming Foam. Environ. Sci. Technol. Lett..

[77] Bolan N. (2019). PFAS beyond defence. Waste + Water Manag. Aust..

[78] Hoisaeter A., Pfaff A., Breedveld G.D. (2019). Leaching and transport of PFAS from aqueous film-forming foam (AFFF) in the unsaturated soil at a firefighting training facility under cold climatic conditions. J. Contam. Hydrol..

[79] Maizel A.C., Shea S., Nickerson A., Schaefer C., Higgins C.P. (2021). Release of Per- and Polyfluoroalkyl Substances from Aqueous Film-Forming Foam Impacted Soils. Environ. Sci. Technol..

[80] Leeson A., Thompson T., Stroo H.F., Anderson R.H., Speicher J., Mills M.A., Willey J., Coyle C., Ghosh R., Lebron C. (2021). Identifying and Managing Aqueous Film-Forming Foam-Derived Per- and Polyfluoroalkyl Substances in the Environment. Environ. Toxicol. Chem..

[81] Johnson G.R. (2022). PFAS in soil and groundwater following historical land application of biosolids. Water Res..

[82] Bolan N., Sarkar B., Vithanage M., Singh G., Tsang D.C.W., Mukhopadhyay R., Ramadass K., Vinu A., Sun Y., Ramanayaka S. (2021). Distribution, behaviour, bioavailability and remediation of poly- and per-fluoroalkyl substances (PFAS) in solid biowastes and biowaste-treated soil. Environ. Int..

[83] Ghisi R., Vamerali T., Manzetti S. (2019). Accumulation of perfluorinated alkyl substances (PFAS) in agricultural plants: A review. Environ. Res..

[84] Wang W., Rhodes G., Ge J., Yu X., Li H. (2020). Uptake and accumulation of per- and polyfluoroalkyl substances in plants. Chemosphere.

[85] Jha G., Kankarla V., McLennon E., Pal S., Sihi D., Dari B., Diaz D., Nocco M. (2021). Per- and Polyfluoroalkyl Substances (PFAS) in Integrated Crop-Livestock Systems: Environmental Exposure and Human Health Risks. Int. J. Environ. Res. Public Health.

[86] Fenton S.E., Ducatman A., Boobis A., DeWitt J.C., Lau C., Ng C., Smith J.S., Roberts S.M. (2021). Per- and Polyfluoroalkyl Substance Toxicity and Human Health Review: Current State of Knowledge and Strategies for Informing Future Research. Environ. Toxicol. Chem..

[87] Adu O., Ma X., Sharma V.K. (2023). Bioavailability, phytotoxicity and plant uptake of per-and polyfluoroalkyl substances (PFAS): A review. J. Hazard. Mater..

[88] Gobelius L., Lewis J., Ahrens L. (2017). Plant Uptake of Per- and Polyfluoroalkyl Substances at a Contaminated Fire Training Facility to Evaluate the Phytoremediation Potential of Various Plant Species. Environ. Sci. Technol..

[89] Baldwin W.S., Davis T.T., Eccles J.A., Patel V.B., Preedy V.R., Rajendram R. (2022). Per- and Polyfluoroalkylsubstances (PFAS) and Their Toxicology as Evidenced through Disease and Biomarkers. Biomarkers in Toxicology.

[90] Langenbach B., Wilson M. (2021). Per- and Polyfluoroalkyl Substances (PFAS): Significance and Considerations within the Regulatory Framework of the USA. Int. J. Environ. Res. Public Health.

[91] Brunn H., Arnold G., Körner W., Rippen G., Steinhäuser K.G., Valentin I. (2023). PFAS: Forever chemicals—Persistent, bioaccumulative and mobile. Reviewing the status and the need for their phase out and remediation of contaminated sites. Environ. Sci. Eur..

[92] Lukić Bilela L., Matijošytė I., Krutkevičius J., Alexandrino D.A.M., Safarik I., Burlakovs J., Gaudêncio S.P., Carvalho M.F. (2023). Impact of per- and polyfluorinated alkyl substances (PFAS) on the marine environment: Raising awareness, challenges, legislation, and mitigation approaches under the One Health concept. Mar. Pollut. Bull..

[93] D’Ambro E.L., Pye H.O.T., Bash J.O., Bowyer J., Allen C., Efstathiou C., Gilliam R.C., Reynolds L., Talgo K., Murphy B.N. (2021). Characterizing the Air Emissions, Transport, and Deposition of Per- and Polyfluoroalkyl Substances from a Fluoropolymer Manufacturing Facility. Environ. Sci. Technol..

[94] Makey C.M., Webster T.F., Martin J.W., Shoeib M., Harner T., Dix-Cooper L., Webster G.M. (2017). Airborne Precursors Predict Maternal Serum Perfluoroalkyl Acid Concentrations. Environ. Sci. Technol..

[95] O’Hagan D. (2008). Understanding organofluorine chemistry. An introduction to the C-F bond. Chem. Soc. Rev..

[96] Cousins I.T., DeWitt J.C., Glüge J., Goldenman G., Herzke D., Lohmann R., Ng C.A., Scheringer M., Wang Z. (2020). The high persistence of PFAS is sufficient for their management as a chemical class. Environ. Sci. Process. Impacts.

[97] Thackray C.P., Selin N.E., Young C.J. (2020). A global atmospheric chemistry model for the fate and transport of PFCAs and their precursors. Environ. Sci. Process. Impacts.

[98] Barton C.A., Zarzecki C.J., Russell M.H. (2010). A site-specific screening comparison of modeled and monitored air dispersion and deposition for perfluorooctanoate. J. Air Waste Manag. Assoc..

[99] Chen H., Yao Y., Zhao Z., Wang Y., Wang Q., Ren C., Wang B., Sun H., Alder A.C., Kannan K. (2018). Multimedia Distribution and Transfer of Per- and Polyfluoroalkyl Substances (PFASs) Surrounding Two Fluorochemical Manufacturing Facilities in Fuxin, China. Environ. Sci. Technol..

[100] Lohmann R., Cousins I.T., DeWitt J.C., Glüge J., Goldenman G., Herzke D., Lindstrom A.B., Miller M.F., Ng C.A., Patton S. (2020). Are Fluoropolymers Really of Low Concern for Human and Environmental Health and Separate from Other PFAS?. Environ. Sci. Technol..

[101] Strynar M., Dagnino S., McMahen R., Liang S., Lindstrom A., Andersen E., McMillan L., Thurman M., Ferrer I., Ball C. (2015). Identification of Novel Perfluoroalkyl Ether Carboxylic Acids (PFECAs) and Sulfonic Acids (PFESAs) in Natural Waters Using Accurate Mass Time-of-Flight Mass Spectrometry (TOFMS). Environ. Sci. Technol..

[102] Sun M., Arevalo E., Strynar M., Lindstrom A., Richardson M., Kearns B., Pickett A., Smith C., Knappe D.R.U. (2016). Legacy and Emerging Perfluoroalkyl Substances Are Important Drinking Water Contaminants in the Cape Fear River Watershed of North Carolina. Environ. Sci. Technol. Lett..

[103] Galloway J.E., Moreno A.V.P., Lindstrom A.B., Strynar M.J., Newton S., May A.A., Weavers L.K. (2020). Evidence of Air Dispersion: HFPO-DA and PFOA in Ohio and West Virginia Surface Water and Soil near a Fluoropolymer Production Facility. Environ. Sci. Technol..

[104] Brandsma S.H., Koekkoek J.C., van Velzen M.J.M., de Boer J. (2019). The PFOA substitute GenX detected in the environment near a fluoropolymer manufacturing plant in the Netherlands. Chemosphere.

[105] De Silva A.O., Allard C.N., Spencer C., Webster G.M., Shoeib M. (2012). Phosphorus-containing fluorinated organics: Polyfluoroalkyl phosphoric acid diesters (diPAPs), perfluorophosphonates (PFPAs), and perfluorophosphinates (PFPIAs) in residential indoor dust. Environ. Sci. Technol..

[106] Fraser A.J., Webster T.F., Watkins D.J., Nelson J.W., Stapleton H.M., Calafat A.M., Kato K., Shoeib M., Vieira V.M., McClean M.D. (2012). Polyfluorinated compounds in serum linked to indoor air in office environments. Environ. Sci. Technol..

[107] Srikantia N., Rekha B., Rajeev A., Kalyan S.N. (2009). Endometrioid endometrial adenocarcinoma in a premenopausal woman with multiple organ metastases. Indian J. Med. Paediatr. Oncol..

[108] Morice P., Leary A., Creutzberg C., Abu-Rustum N., Darai E. (2016). Endometrial cancer. Lancet.

[109] Ferlay J., Soerjomataram I., Dikshit R., Eser S., Mathers C., Rebelo M., Parkin D.M., Forman D., Bray F. (2015). Cancer incidence and mortality worldwide: Sources, methods and major patterns in GLOBOCAN 2012. Int. J. Cancer.

[110] Raglan O., Kalliala I., Markozannes G., Cividini S., Gunter M.J., Nautiyal J., Gabra H., Paraskevaidis E., Martin-Hirsch P., Tsilidis K.K. (2019). Risk factors for endometrial cancer: An umbrella review of the literature. Int. J. Cancer.

[111] Lortet-Tieulent J., Ferlay J., Bray F., Jemal A. (2018). International Patterns and Trends in Endometrial Cancer Incidence, 1978–2013. J. Natl. Cancer Inst..

[112] Gentry-Maharaj A., Karpinskyj C. (2020). Current and future approaches to screening for endometrial cancer. Best Pract. Res. Clin. Obstet. Gynaecol..

[113] Sanderson P.A., Critchley H.O.D., Williams A.R.W., Arends M.J., Saunders P.T.K. (2016). New concepts for an old problem: The diagnosis of endometrial hyperplasia. Hum. Reprod. Update.

[114] Setiawan V.W., Yang H.P., Pike M.C., McCann S.E., Yu H., Xiang Y.B., Wolk A., Wentzensen N., Weiss N.S., Webb P.M. (2013). Type I and II endometrial cancers: Have they different risk factors?. J. Clin. Oncol..

[115] Yang H.P., Wentzensen N., Trabert B., Gierach G.L., Felix A.S., Gunter M.J., Hollenbeck A., Park Y., Sherman M.E., Brinton L.A. (2013). Endometrial cancer risk factors by 2 main histologic subtypes: The NIH-AARP Diet and Health Study. Am. J. Epidemiol..

[116] Ebring C., Marlin R., Macni J., Vallard A., Bergerac S., Beaubrun-Renard M., Joachim C., Jean-Laurent M. (2023). Type II endometrial cancer: Incidence, overall and disease-free survival in Martinique. PLoS ONE.

[117] Talhouk A., McAlpine J.N. (2016). New classification of endometrial cancers: The development and potential applications of genomic-based classification in research and clinical care. Gynecol. Oncol. Res. Pract..

[118] Bruggmann D., Ouassou K., Klingelhofer D., Bohlmann M.K., Jaque J., Groneberg D.A. (2020). Endometrial cancer: Mapping the global landscape of research. J. Transl. Med..

[119] Njoku K., Abiola J., Russell J., Crosbie E.J. (2020). Endometrial cancer prevention in high-risk women. Best Pract. Res. Clin. Obstet. Gynaecol..

[120] Kalampokas E., Giannis G., Kalampokas T., Papathanasiou A.-A., Mitsopoulou D., Tsironi E., Triantafyllidou O., Gurumurthy M., Parkin D.E., Cairns M. (2022). Current approaches to the management of patients with endometrial cancer. Cancers.

[121] Purdie D.M., Green A.C. (2001). Epidemiology of endometrial cancer. Best Pract. Res. Clin. Obstet. Gynaecol..

[122] Semple D. (1997). Endometrial cancer. Br. J. Hosp. Med..

[123] Landis S.H., Murray T., Bolden S., Wingo P.A. (1999). Cancer statistics, 1999. CA A Cancer J. Clin..

[124] Donkers H., Bekkers R., Galaal K. (2020). Diagnostic value of microRNA panel in endometrial cancer: A systematic review. Oncotarget.

[125] Randall M.E., Filiaci V.L., Muss H., Spirtos N.M., Mannel R.S., Fowler J., Thigpen J.T., Benda J.A. (2006). Randomized phase III trial of whole-abdominal irradiation versus doxorubicin and cisplatin chemotherapy in advanced endometrial carcinoma: A Gynecologic Oncology Group Study. J. Clin. Oncol..

[126] Keys H.M., Roberts J.A., Brunetto V.L., Zaino R.J., Spirtos N.M., Bloss J.D., Pearlman A., Maiman M.A., Bell J.G. (2004). A phase III trial of surgery with or without adjunctive external pelvic radiation therapy in intermediate risk endometrial adenocarcinoma: A Gynecologic Oncology Group study. Gynecol. Oncol..

[127] Santin A.D., Bellone S., O’Brien T.J., Pecorelli S., Cannon M.J., Roman J.J. (2004). Current treatment options for endometrial cancer. Expert Rev. Anticancer. Ther..

[128] Hogberg T., Signorelli M., de Oliveira C.F., Fossati R., Lissoni A.A., Sorbe B., Andersson H., Grenman S., Lundgren C., Rosenberg P. (2010). Sequential adjuvant chemotherapy and radiotherapy in endometrial cancer—Results from two randomised studies. Eur. J. Cancer.

[129] Brown K.F., Rumgay H., Dunlop C., Ryan M., Quartly F., Cox A., Deas A., Elliss-Brookes L., Gavin A., Hounsome L. (2018). The fraction of cancer attributable to modifiable risk factors in England, Wales, Scotland, Northern Ireland, and the United Kingdom in 2015. Br. J. Cancer.

[130] Tzenios N., Chahine M., Tazanios M. (2023). Obesity and endometrial cancer: The role insulin resistance and adipokines. Spec. J. Med. Acad. Other Life Sci..

[131] Saliha S., Venketeshwer R., Leticia R. (2021). Obesity and Endometrial Cancer. Role of Obesity in Human Health and Disease.

[132] Aune D., Navarro Rosenblatt D.A., Chan D.S.M., Vingeliene S., Abar L., Vieira A.R., Greenwood D.C., Bandera E.V., Norat T. (2015). Anthropometric factors and endometrial cancer risk: A systematic review and dose–response meta-analysis of prospective studies. Ann. Oncol..

[133] Mackintosh M.L., Crosbie E.J. (2013). Obesity-driven endometrial cancer: Is weight loss the answer?. BJOG.

[134] Adams T.D., Gress R.E., Smith S.C., Halverson R.C., Simper S.C., Rosamond W.D., LaMonte M.J., Stroup A.M., Hunt S.C. (2007). Long-Term Mortality after Gastric Bypass Surgery. N. Engl. J. Med..

[135] Ignatov A., Ortmann O. (2020). Endocrine Risk Factors of Endometrial Cancer: Polycystic Ovary Syndrome, Oral Contraceptives, Infertility, Tamoxifen. Cancers.

[136] Prakash A., Nourianpour M., Senok A., Atiomo W. (2022). Polycystic Ovary Syndrome and Endometrial Cancer: A Scoping Review of the Literature on Gut Microbiota. Cells.

[137] Che X., Jian F., Chen C., Liu C., Liu G., Feng W. (2020). PCOS serum-derived exosomal miR-27a-5p stimulates endometrial cancer cells migration and invasion. J. Mol. Endocrinol..

[138] Grady D., Gebretsadik T., Kerlikowske K., Ernster V., Petitti D. (1995). Hormone replacement therapy and endometrial cancer risk: A meta-analysis. Obstet. Gynecol..

[139] Lees B., Hampton J.M., Trentham-Dietz A., Newcomb P., Spencer R. (2021). A population-based study of causes of death after endometrial cancer according to major risk factors. Gynecol. Oncol..

[140] Zhang Z.-H., Su P.-Y., Hao J.-H., Sun Y.-H. (2013). The Role of Preexisting Diabetes Mellitus on Incidence and Mortality of Endometrial Cancer: A Meta-Analysis of Prospective Cohort Studies. Int. J. Gynecol. Cancer.

[141] Emons G., Mustea A., Tempfer C. (2020). Tamoxifen and Endometrial Cancer: A Janus-Headed Drug. Cancers.

[142] Choi S., Lee Y.J., Jeong J.H., Jung J., Lee J.W., Kim H.J., Ko B.S., Son B.H., Ahn S.H., Lee Y. (2021). Risk of Endometrial Cancer and Frequencies of Invasive Endometrial Procedures in Young Breast Cancer Survivors Treated With Tamoxifen: A Nationwide Study. Front. Oncol..

[143] Schmid D., Behrens G., Keimling M., Jochem C., Ricci C., Leitzmann M. (2015). A systematic review and meta-analysis of physical activity and endometrial cancer risk. Eur. J. Epidemiol..

[144] Dimitrios A.K., Rebecca J.B., Ranjit M., Moscho M., Matthew B., Knobf M.T., Anne L. (2017). Recruitment, adherence, and retention of endometrial cancer survivors in a behavioural lifestyle programme: The Diet and Exercise in Uterine Cancer Survivors (DEUS) parallel randomised pilot trial. BMJ Open.

[145] Crous-Bou M., Du M., Gunter M.J., Setiawan V.W., Schouten L.J., Shu X.-o., Wentzensen N., Bertrand K.A., Cook L.S., Friedenreich C.M. (2022). Coffee consumption and risk of endometrial cancer: A pooled analysis of individual participant data in the Epidemiology of Endometrial Cancer Consortium (E2C2). Am. J. Clin. Nutr..

[146] Brooks R.A., Fleming G.F., Lastra R.R., Lee N.K., Moroney J.W., Son C.H., Tatebe K., Veneris J.L. (2019). Current recommendations and recent progress in endometrial cancer. CA A Cancer J. Clin..

[147] Temkin S.M., Kohn E.C., Penberthy L., Cronin K.A., Rubinsak L., Dickie L.A., Minasian L., Noone A.-M. (2018). Hysterectomy-corrected rates of endometrial cancer among women younger than age 50 in the United States. Cancer Causes Control.

[148] Siegel R., Naishadham D., Jemal A. (2013). Cancer statistics, 2013. CA A Cancer J. Clin..

[149] Levine D.A., Getz G., Gabriel S.B., Cibulskis K., Lander E., Sivachenko A., Sougnez C., Lawrence M., Kandoth C., Dooling D. (2013). Integrated genomic characterization of endometrial carcinoma. Nature.

[150] Constantine G.D., Kessler G., Graham S., Goldstein S.R. (2019). Increased Incidence of Endometrial Cancer Following the Women’s Health Initiative: An Assessment of Risk Factors. J. Womens Health.

[151] Wartko P., Sherman M.E., Yang H.P., Felix A.S., Brinton L.A., Trabert B. (2013). Recent changes in endometrial cancer trends among menopausal-age U.S. women. Cancer Epidemiol..

[152] Rossouw J.E., Anderson G.L., Prentice R.L., LaCroix A.Z., Kooperberg C., Stefanick M.L., Jackson R.D., Beresford S.A., Howard B.V., Johnson K.C. (2002). Risks and benefits of estrogen plus progestin in healthy postmenopausal women: Principal results From the Women’s Health Initiative randomized controlled trial. JAMA.

[153] Passarello K., Kurian S., Villanueva V. (2019). Endometrial Cancer: An Overview of Pathophysiology, Management, and Care. Semin. Oncol. Nurs..

[154] Nelson J.W., Scammell M.K., Hatch E.E., Webster T.F. (2012). Social disparities in exposures to bisphenol A and polyfluoroalkyl chemicals: A cross-sectional study within NHANES 2003–2006. Environ. Health.

[155] Kato K., Wong L.Y., Chen A., Dunbar C., Webster G.M., Lanphear B.P., Calafat A.M. (2014). Changes in serum concentrations of maternal poly- and perfluoroalkyl substances over the course of pregnancy and predictors of exposure in a multiethnic cohort of Cincinnati, Ohio pregnant women during 2003–2006. Environ. Sci. Technol..

[156] Buekers J., Colles A., Cornelis C., Morrens B., Govarts E., Schoeters G. (2018). Socio-economic status and health: Evaluation of human biomonitored chemical exposure to per-and polyfluorinated substances across status. Int. J. Environ. Res. Public Health.

[157] Kingsley S.L., Eliot M.N., Kelsey K.T., Calafat A.M., Ehrlich S., Lanphear B.P., Chen A., Braun J.M. (2018). Variability and predictors of serum perfluoroalkyl substance concentrations during pregnancy and early childhood. Environ. Res..

[158] Kato K., Wong L.Y., Jia L.T., Kuklenyik Z., Calafat A.M. (2011). Trends in exposure to polyfluoroalkyl chemicals in the U.S. Population: 1999–2008. Environ. Sci. Technol..

[159] Uhl S.A., James-Todd T., Bell M.L. (2013). Association of Osteoarthritis with Perfluorooctanoate and Perfluorooctane Sulfonate in NHANES 2003–2008. Environ. Health Perspect..

[160] Obeng-Gyasi E. (2022). Factors associated with elevated Per- and Polyfluoroalkyl substances serum levels in older adults. Aging Health Res..

[161] Chang C.J., Ryan P.B., Smarr M.M., Kannan K., Panuwet P., Dunlop A.L., Corwin E.J., Barr D.B. (2021). Serum per- and polyfluoroalkyl substance (PFAS) concentrations and predictors of exposure among pregnant African American women in the Atlanta area, Georgia. Environ. Res..

[162] Eick S.M., Enright E.A., Geiger S.D., Dzwilewski K.L.C., DeMicco E., Smith S., Park J.S., Aguiar A., Woodruff T.J., Morello-Frosch R. (2021). Associations of Maternal Stress, Prenatal Exposure to Per- and Polyfluoroalkyl Substances (PFAS), and Demographic Risk Factors with Birth Outcomes and Offspring Neurodevelopment: An Overview of the ECHO.CA.IL Prospective Birth Cohorts. Int. J. Environ. Res. Public Health.

[163] Wise L.A., Wesselink A.K., Schildroth S., Calafat A.M., Bethea T.N., Geller R.J., Coleman C.M., Fruh V., Claus Henn B., Botelho J.C. (2022). Correlates of plasma concentrations of per- and poly-fluoroalkyl substances among reproductive-aged Black women. Environ. Res..

[164] Sagiv S.K., Rifas-Shiman S.L., Webster T.F., Mora A.M., Harris M.H., Calafat A.M., Ye X., Gillman M.W., Oken E. (2015). Sociodemographic and Perinatal Predictors of Early Pregnancy Per- and Polyfluoroalkyl Substance (PFAS) Concentrations. Environ. Sci. Technol..

[165] Lung F.W., Chiang T.L., Lin S.J., Lee M.C., Shu B.C. (2018). Advanced Maternal Age and Maternal Education Disparity in Children with Autism Spectrum Disorder. Matern. Child. Health J..

[166] Torvik F.A., Eilertsen E.M., McAdams T.A., Gustavson K., Zachrisson H.D., Brandlistuen R., Gjerde L.C., Havdahl A., Stoltenberg C., Ask H. (2020). Mechanisms linking parental educational attainment with child ADHD, depression, and academic problems: A study of extended families in The Norwegian Mother, Father and Child Cohort Study. J. Child Psychol. Psychiatry.

[167] Skogheim T.S., Weyde K.V.F., Aase H., Engel S.M., Suren P., Oie M.G., Biele G., Reichborn-Kjennerud T., Brantsaeter A.L., Haug L.S. (2021). Prenatal exposure to per- and polyfluoroalkyl substances (PFAS) and associations with attention-deficit/hyperactivity disorder and autism spectrum disorder in children. Environ. Res..

[168] Montazeri P., Thomsen C., Casas M., de Bont J., Haug L.S., Maitre L., Papadopoulou E., Sakhi A.K., Slama R., Saulnier P.J. (2019). Socioeconomic position and exposure to multiple environmental chemical contaminants in six European mother-child cohorts. Int. J. Hyg. Environ. Health.

[169] Dalsager L., Jensen T.K., Nielsen F., Grandjean P., Bilenberg N., Andersen H.R. (2021). No association between maternal and child PFAS concentrations and repeated measures of ADHD symptoms at age 2(1/2) and 5 years in children from the Odense Child Cohort. Neurotoxicol. Teratol..

[170] Touvier M., Kesse-Guyot E., Méjean C., Estaquio C., Péneau S., Hercberg S., Castetbon K. (2010). Variations in Compliance with Recommendations and Types of Meat/Seafood/Eggs according to Sociodemographic and Socioeconomic Categories. Ann. Nutr. Metab..

[171] Menzel J., Abraham K., Dietrich S., Fromme H., Volkel W., Schwerdtle T., Weikert C. (2021). Internal exposure to perfluoroalkyl substances (PFAS) in vegans and omnivores. Int. J. Hyg. Environ. Health.

[172] Lucas K., Gaines L.G.T., Paris-Davila T., Nylander-French L.A. (2022). Occupational exposure and serum levels of per- and polyfluoroalkyl substances (PFAS): A review. Am. J. Ind. Med..

[173] Paris-Davila T., Gaines L.G.T., Lucas K., Nylander-French L.A. (2023). Occupational exposures to airborne per- and polyfluoroalkyl substances (PFAS)—A review. Am. J. Ind. Med..

[174] Gaines L.G.T. (2022). Historical and current usage of per- and polyfluoroalkyl substances (PFAS): A literature review. Am. J. Ind. Med..

[175] Peaslee G.F., Wilkinson J.T., McGuinness S.R., Tighe M., Caterisano N., Lee S., Gonzales A., Roddy M., Mills S., Mitchell K. (2020). Another Pathway for Firefighter Exposure to Per- and Polyfluoroalkyl Substances: Firefighter Textiles. Environ. Sci. Technol. Lett..

[176] Leary D.B., Takazawa M., Kannan K., Khalil N. (2020). Perfluoroalkyl Substances and Metabolic Syndrome in Firefighters: A Pilot Study. J. Occup. Environ. Med..

[177] Freberg B.I., Haug L.S., Olsen R., Daae H.L., Hersson M., Thomsen C., Thorud S., Becher G., Molander P., Ellingsen D.G. (2010). Occupational Exposure to Airborne Perfluorinated Compounds during Professional Ski Waxing. Environ. Sci. Technol..

[178] Nilsson H., Kärrman A., Westberg H., Rotander A., van Bavel B., Lindström G. (2010). A Time Trend Study of Significantly Elevated Perfluorocarboxylate Levels in Humans after Using Fluorinated Ski Wax. Environ. Sci. Technol..

[179] Crawford K.A., Doherty B.T., Gilbert-Diamond D., Romano M.E., Claus Henn B. (2022). Waxing activity as a potential source of exposure to per- and polyfluoroalkyl substances (PFAS) and other environmental contaminants among the US ski and snowboard community. Environ. Res..

[180] Glynn A., Berger U., Bignert A., Ullah S., Aune M., Lignell S., Darnerud P.O. (2012). Perfluorinated Alkyl Acids in Blood Serum from Primiparous Women in Sweden: Serial Sampling during Pregnancy and Nursing, And Temporal Trends 1996–2010. Environ. Sci. Technol..

[181] Stubleski J., Salihovic S., Lind P.M., Lind L., Dunder L., McCleaf P., Euren K., Ahrens L., Svartengren M., van Bavel B. (2017). The effect of drinking water contaminated with perfluoroalkyl substances on a 10-year longitudinal trend of plasma levels in an elderly Uppsala cohort. Environ. Res..

[182] Waterfield G., Rogers M., Grandjean P., Auffhammer M., Sunding D. (2020). Reducing exposure to high levels of perfluorinated compounds in drinking water improves reproductive outcomes: Evidence from an intervention in Minnesota. Environ. Health.

[183] Morgan S., Mottaleb M.A., Kraemer M.P., Moser D.K., Worley J., Morris A.J., Petriello M.C. (2023). Effect of lifestyle-based lipid lowering interventions on the relationship between circulating levels of per-and polyfluoroalkyl substances and serum cholesterol. Environ. Toxicol. Pharmacol..

[184] Hurley S., Goldberg D., Wang M., Park J.S., Petreas M., Bernstein L., Anton-Culver H., Nelson D.O., Reynolds P. (2018). Time Trends in Per- and Polyfluoroalkyl Substances (PFASs) in California Women: Declining Serum Levels, 2011–2015. Environ. Sci. Technol..

[185] Blake B.E., Pinney S.M., Hines E.P., Fenton S.E., Ferguson K.K. (2018). Associations between longitudinal serum perfluoroalkyl substance (PFAS) levels and measures of thyroid hormone, kidney function, and body mass index in the Fernald Community Cohort. Environ. Pollut..

[186] Ward-Caviness C.K., Moyer J., Weaver A., Devlin R., Diaz-Sanchez D. (2022). Associations between PFAS occurrence and multimorbidity as observed in an electronic health record cohort. Environ. Epidemiol..

[187] Liu Z., Yang J.Z. (2023). Communicating Per- and Polyfluoroalkyl Substances (PFAS) Contamination to the Public Through Personal Relevance. J. Health Commun..

[188] Lin P.D., Cardenas A., Hauser R., Gold D.R., Kleinman K.P., Hivert M.F., Calafat A.M., Webster T.F., Horton E.S., Oken E. (2020). Per- and polyfluoroalkyl substances and blood pressure in pre-diabetic adults—Cross-sectional and longitudinal analyses of the diabetes prevention program outcomes study. Environ. Int..

[189] Pitter G., Zare Jeddi M., Barbieri G., Gion M., Fabricio A.S.C., Dapra F., Russo F., Fletcher T., Canova C. (2020). Perfluoroalkyl substances are associated with elevated blood pressure and hypertension in highly exposed young adults. Environ. Health.

[190] Boronow K.E., Brody J.G., Schaider L.A., Peaslee G.F., Havas L., Cohn B.A. (2019). Serum concentrations of PFASs and exposure-related behaviors in African American and non-Hispanic white women. J. Expo. Sci. Environ. Epidemiol..

[191] Ding N., Harlow S.D., Batterman S., Mukherjee B., Park S.K. (2020). Longitudinal trends in perfluoroalkyl and polyfluoroalkyl substances among multiethnic midlife women from 1999 to 2011: The Study of Women’s Health Across the Nation. Environ. Int..

[192] Karia P.S., Huang Y., Tehranifar P., Wright J.D., Genkinger J.M. (2023). Racial and ethnic differences in type II endometrial cancer mortality outcomes: The contribution of sociodemographic, clinicopathologic, and treatment factors. Gynecol. Oncol..

[193] Suarez A.A., Felix A.S., Cohn D.E. (2017). Bokhman Redux: Endometrial cancer “types” in the 21st century. Gynecol. Oncol..

[194] Benoit L., Pauly L., Phelippeau J., Koskas M. (2020). Impact of Sociodemographic Characteristics on the Quality of Care in the Surgical Management of Endometrial Cancer: An Analysis of a National Database in the United States. Gynecol. Obstet. Investig..

[195] Rodriguez V.E., LeBrón A.M.W., Chang J., Bristow R.E. (2021). Guideline-adherent treatment, sociodemographic disparities, and cause-specific survival for endometrial carcinomas. Cancer.

[196] Wang S.M., Moore C., Keegan E., Mayer C., Litman E., Das K.J.H., Wu C.Z., Chappell N.P. (2023). Analysis of Sociodemographic Factors Affecting Ambulatory Surgical Center Discharge Patterns for Endometrial Cancer Hysterectomies. J. Minim. Invasive Gynecol..

[197] Zheng W. (2023). Molecular Classification of Endometrial Cancer and the 2023 FIGO Staging: Exploring the Challenges and Opportunities for Pathologists. Cancers.

[198] Hill H.A., Eley J.W., Harlan L.C., Greenberg R.S., Barrett II R.J., Chen V.W. (1996). Racial differences in endometrial cancer survival: The black/white cancer survival study. Obstet. Gynecol..

[199] Chan J.K., Sherman A.E., Kapp D.S., Zhang R., Osann K.E., Maxwell L., Chen L.M., Deshmukh H. (2011). Influence of gynecologic oncologists on the survival of patients with endometrial cancer. J. Clin. Oncol..

[200] Francies F.Z., Marima R., Hull R., Molefi T., Dlamini Z. (2020). Genomics and splicing events of type II endometrial cancers in the black population: Racial disparity, socioeconomic and geographical differences. Am. J. Cancer Res..

[201] Darin-Mattsson A., Fors S., Kåreholt I. (2017). Different indicators of socioeconomic status and their relative importance as determinants of health in old age. Int. J. Equity Health.

[202] Madison T., Schottenfeld D., James S.A., Schwartz A.G., Gruber S.B. (2004). Endometrial cancer: Socioeconomic status and racial/ethnic differences in stage at diagnosis, treatment, and survival. Am. J. Public Health.

[203] Long B., Liu F.W., Bristow R.E. (2013). Disparities in uterine cancer epidemiology, treatment, and survival among African Americans in the United States. Gynecol. Oncol..

[204] Kucera C.W., Tian C., Tarney C.M., Presti C., Jokajtys S., Winkler S.S., Casablanca Y., Bateman N.W., Mhawech-Fauceglia P., Wenzel L. (2023). Factors Associated With Survival Disparities Between Non-Hispanic Black and White Patients With Uterine Cancer. JAMA Netw. Open.

[205] Hayden J. (2022). Introduction to Health Behavior Theory.

[206] Washington C.R., Haggerty A., Ronner W., Neff P.M., Ko E.M. (2020). Knowledge of endometrial cancer risk factors in a general gynecologic population. Gynecol. Oncol..

[207] Blackburn B.E., Soisson S., Rowe K., Snyder J., Fraser A., Deshmukh V., Newman M., Smith K., Herget K., Kirchhoff A.C. (2019). Prognostic factors for rural endometrial cancer patients in a population-based cohort. BMC Public Health.

[208] Wernli K.J., Ray R.M., Gao D.L., Fitzgibbons E.D., Camp J.E., Astrakianakis G., Seixas N., Li W., De Roos A.J., Feng Z. (2008). Occupational risk factors for endometrial cancer among textile workers in Shanghai, China. Am. J. Ind. Med..

[209] Weiderpass E., Pukkala E., Vasama-Neuvonen K., Kauppinen T., Vainio H., Paakkulainen H., Boffetta P., Partanen T. (2001). Occupational exposures and cancers of the endometrium and cervix uteri in Finland. Am. J. Ind. Med..

[210] Mallozzi M., Leone C., Manurita F., Bellati F., Caserta D. (2017). Endocrine Disrupting Chemicals and Endometrial Cancer: An Overview of Recent Laboratory Evidence and Epidemiological Studies. Int. J. Environ. Res. Public Health.

[211] Caserta D., De Marco M.P., Besharat A.R., Costanzi F. (2022). Endocrine Disruptors and Endometrial Cancer: Molecular Mechanisms of Action and Clinical Implications, a Systematic Review. Int. J. Mol. Sci..

[212] Khan S., Duan P., Yao L., Hou H. (2018). Shiftwork-Mediated Disruptions of Circadian Rhythms and Sleep Homeostasis Cause Serious Health Problems. Int. J. Genom..

[213] Ball L.J., Palesh O., Kriegsfeld L.J. (2016). The Pathophysiologic Role of Disrupted Circadian and Neuroendocrine Rhythms in Breast Carcinogenesis. Endocr. Rev..

[214] Wang Z., Wang H., Wang Z., He S., Jiang Z., Yan C., Zhang S., Wang T. (2020). Associated analysis of PER1/TUBB2B with endometrial cancer development caused by circadian rhythm disorders. Med. Oncol..

[215] Kravchenko J., Akushevich I., Rhew S.H., Agarwal P., Lyerly H.K. (2020). Uterine Cancer Mortality in White and African American Females in Southeastern North Carolina. J. Environ. Public Health.

[216] Dutta S., Gorain B., Choudhury H., Roychoudhury S., Sengupta P. (2022). Environmental and occupational exposure of metals and female reproductive health. Environ. Sci. Pollut. Res..

[217] Barrington D.A., Sinnott J.A., Calo C., Cohn D.E., Cosgrove C.M., Felix A.S. (2020). Where you live matters: A National Cancer Database study of Medicaid expansion and endometrial cancer outcomes. Gynecol. Oncol..

[218] Albright B.B., Nasioudis D., Craig S., Moss H.A., Latif N.A., Ko E.M., Haggerty A.F. (2021). Impact of Medicaid expansion on women with gynecologic cancer: A difference-in-difference analysis. Am. J. Obstet. Gynecol..

[219] Kaspers M., Llamocca E., Quick A., Dholakia J., Salani R., Felix A.S. (2020). Black and Hispanic women are less likely than white women to receive guideline-concordant endometrial cancer treatment. Am. J. Obstet. Gynecol..

[220] Straubhar A.M., Parsons M.W., Francis S., Gaffney D., Maurer K.A. (2021). Refusal of surgery and survival outcomes in endometrial cancer. Int. J. Gynecol. Cancer.

[221] Barrington D.A., Sinnott J.A., Nixon D., Padamsee T.J., Cohn D.E., Doll K.M., Donneyong M.M., Felix A.S. (2022). More than treatment refusal: A National Cancer Database analysis of adjuvant treatment refusal and racial survival disparities among women with endometrial cancer. Am. J. Obstet. Gynecol..

[222] Gould J.M., Marlin A.T. (2019). Quality of Life in American Neighborhoods: Levels of Affluence, Toxic Waste, and Cancer Mortality in Residential Zip Code Areas.

[223] Rodriguez V.E., LeBrón A.M., Chang J., Bristow R.E. (2021). Racial–ethnic and socioeconomic disparities in guideline-adherent treatment for endometrial cancer. Obstet. Gynecol..

[224] Setiawan V.W., Pike M.C., Kolonel L.N., Nomura A.M., Goodman M.T., Henderson B.E. (2007). Racial/ethnic differences in endometrial cancer risk: The multiethnic cohort study. Am. J. Epidemiol..

[225] Liu L., Habeshian T.S., Zhang J., Peeri N.C., Du M., De Vivo I., Setiawan V.W. (2023). Differential trends in rising endometrial cancer incidence by age, race, and ethnicity. JNCI Cancer Spectr..

[226] Siegel R.L., Miller K.D., Fuchs H.E., Jemal A. (2022). Cancer statistics, 2022. CA A Cancer J. Clin..

[227] Giaquinto A.N., Broaddus R.R., Jemal A., Siegel R.L. (2022). The Changing Landscape of Gynecologic Cancer Mortality in the United States. Obstet. Gynecol..

[228] Desmond D., Arter Z., Berenberg J.L., Killeen J.L., Bunch K., Merritt M.A. (2023). Racial and ethnic differences in tumor characteristics among endometrial cancer patients in an equal-access healthcare population. Cancer Causes Control.

[229] Polymeros K., Guttery D.S., Hew R., Bishop R., Stannard E., Macip S., Symonds P., Moss E.L. (2020). Differences in the molecular profile of endometrial cancers from British White and British South Asian women. PLoS ONE.

[230] Leung Y.-K. (2023). A Silent Threat: Exploring the Impact of Endocrine Disruption on Human Health. Int. J. Mol. Sci..

[231] Hassan S., Thacharodi A., Priya A., Meenatchi R., Hegde T.A., Thangamani R., Nguyen H.T., Pugazhendhi A. (2024). Endocrine disruptors: Unravelling the link between chemical exposure and Women’s reproductive health. Environ. Res..

[232] Averina M., Huber S., Almås B., Brox J., Jacobsen B.K., Furberg A.-S., Grimnes G. (2024). Early menarche and other endocrine disrupting effects of per- and polyfluoroalkyl substances (PFAS) in adolescents from Northern Norway. The Fit Futures study. Environ. Res..

[233] Yi W., Xuan L., Zakaly H.M.H., Markovic V., Miszczyk J., Guan H., Zhou P.-K., Huang R. (2023). Association between per- and polyfluoroalkyl substances (PFAS) and depression in U.S. adults: A cross-sectional study of NHANES from 2005 to 2018. Environ. Res..

[234] Yasin H.K., Taylor A.H., Ayakannu T. (2021). A Narrative Review of the Role of Diet and Lifestyle Factors in the Development and Prevention of Endometrial Cancer. Cancers.

[235] Eick S.M., Barr D.B., Brennan P.A., Taibl K.R., Tan Y., Robinson M., Kannan K., Panuwet P., Yakimavets V., Ryan P.B. (2023). Per- and polyfluoroalkyl substances and psychosocial stressors have a joint effect on adverse pregnancy outcomes in the Atlanta African American Maternal-Child cohort. Sci. Total Environ..

[236] Ma Z., Liu X., Li F., Wang Y., Xu Y., Zhang M., Zhang X., Ying X., Zhang X. (2016). Perfluorooctanoic acid induces human Ishikawa endometrial cancer cell migration and invasion through activation of ERK/mTOR signaling. Oncotarget.

[237] Boafo Y.S., Mostafa S., Obeng-Gyasi E. (2023). Association of Combined Metals and PFAS with Cardiovascular Disease Risk. Toxics.

[238] Zhang Y., Zhang L., Bao J., Liu L., Wang X. (2021). Perfluorooctanoic acid exposure in early pregnancy induces oxidative stress in mice uterus and liver. Environ. Sci. Pollut. Res..

[239] Coperchini F., Croce L., Ricci G., Magri F., Rotondi M., Imbriani M., Chiovato L. (2021). Thyroid disrupting effects of old and new generation PFAS. Front. Endocrinol..

[240] Toporova L., Balaguer P. (2020). Nuclear receptors are the major targets of endocrine disrupting chemicals. Mol. Cell. Endocrinol..

[241] Watkins D.J., Wellenius G.A., Butler R.A., Bartell S.M., Fletcher T., Kelsey K.T. (2014). Associations between serum perfluoroalkyl acids and LINE-1 DNA methylation. Environ. Int..

[242] Guerrero-Preston R., Goldman L.R., Brebi-Mieville P., Ili-Gangas C., LeBron C., Witter F.R., Apelberg B.J., Hernández-Roystacher M., Jaffe A., Halden R.U. (2010). Global DNA hypomethylation is associated with in utero exposure to cotinine and perfluorinated alkyl compounds. Epigenetics.

[243] Boyd R.I., Ahmad S., Singh R., Fazal Z., Prins G.S., Madak Erdogan Z., Irudayaraj J., Spinella M.J. (2022). Toward a mechanistic understanding of poly-and perfluoroalkylated substances and cancer. Cancers.

[244] Tian M., Peng S., Martin F.L., Zhang J., Liu L., Wang Z., Dong S., Shen H. (2012). Perfluorooctanoic acid induces gene promoter hypermethylation of glutathione-S-transferase Pi in human liver L02 cells. Toxicology.

[245] Liu C.-Y., Chen P.-C., Lien P.-C., Liao Y.-P. (2018). Prenatal perfluorooctyl sulfonate exposure and Alu DNA hypomethylation in cord blood. Int. J. Environ. Res. Public Health.

[246] Di Nisio A., Rocca M., Sabovic I., Ponce M.D.R., Corsini C., Guidolin D., Zanon C., Acquasaliente L., Carosso A., De Toni L. (2020). Perfluorooctanoic acid alters progesterone activity in human endometrial cells and induces reproductive alterations in young women. Chemosphere.

[247] Charazac A., Hinault C., Dolfi B., Hautier S., Decondé Le Butor C., Bost F., Chevalier N. (2022). Low Doses of PFOA Promote Prostate and Breast Cancer Cells Growth through Different Pathways. Int. J. Mol. Sci..

[248] Li X., Bao C., Ma Z., Xu B., Ying X., Liu X., Zhang X. (2018). Perfluorooctanoic acid stimulates ovarian cancer cell migration, invasion via ERK/NF-κB/MMP-2/-9 pathway. Toxicol. Lett..

[249] Cathey A.L., Nguyen V.K., Colacino J.A., Woodruff T.J., Reynolds P., Aung M.T. (2023). Exploratory profiles of phenols, parabens, and per- and poly-fluoroalkyl substances among NHANES study participants in association with previous cancer diagnoses. J. Expo. Sci. Environ. Epidemiol..

[250] Li H., Hammarstrand S., Midberg B., Xu Y., Li Y., Olsson D.S., Fletcher T., Jakobsson K., Andersson E.M. (2022). Cancer incidence in a Swedish cohort with high exposure to perfluoroalkyl substances in drinking water. Environ. Res..

[251] Leonard R.C., Kreckmann K.H., Sakr C.J., Symons J.M. (2008). Retrospective Cohort Mortality Study of Workers in a Polymer Production Plant Including a Reference Population of Regional Workers. Ann. Epidemiol..

[252] Law H.D., Armstrong B.K., D’este C., Hosking R., Smurthwaite K.S., Trevenar S., Lucas R.M., Lazarevic N., Kirk M.D., Korda R.J. (2023). Relative rates of cancers and deaths in Australian communities with PFAS environmental contamination associated with firefighting foams: A cohort study using linked data. Cancer Epidemiol..

[253] Grandjean P., Clapp R. (2015). Perfluorinated Alkyl Substances: Emerging Insights Into Health Risks. New Solut..

[254] Gilliland F.D., Mandel J.S. (1993). Mortality among employees of a perfluorooctanoic acid production plant. J. Occup. Med..

[255] Maddela N.R., Ramakrishnan B., Kakarla D., Venkateswarlu K., Megharaj M. (2022). Major contaminants of emerging concern in soils: A perspective on potential health risks. RSC Adv..

[256] Imir O.B., Kaminsky A.Z., Zuo Q.-Y., Liu Y.-J., Singh R., Spinella M.J., Irudayaraj J., Hu W.-Y., Prins G.S., Madak Erdogan Z. (2021). Per- and Polyfluoroalkyl Substance Exposure Combined with High-Fat Diet Supports Prostate Cancer Progression. Nutrients.

[257] Campbell S., Raza M., Pollack A.Z. (2016). Perfluoroalkyl substances and endometriosis in US women in NHANES 2003–2006. Reprod. Toxicol..

[258] Chaney C., Wiley K.S. (2023). The variable associations between PFASs and biological aging by sex and reproductive stage in NHANES 1999–2018. Environ. Res..

[259] Padula A.M., Ning X., Bakre S., Barrett E.S., Bastain T., Bennett D.H., Bloom M.S., Breton C.V., Dunlop A.L., Eick S.M. (2023). Birth Outcomes in Relation to Prenatal Exposure to Per- and Polyfluoroalkyl Substances and Stress in the Environmental Influences on Child Health Outcomes (ECHO) Program. Environ. Health Perspect..

[260] Eick S.M., Goin D.E., Cushing L., DeMicco E., Smith S., Park J.-S., Padula A.M., Woodruff T.J., Morello-Frosch R. (2022). Joint effects of prenatal exposure to per- and poly-fluoroalkyl substances and psychosocial stressors on corticotropin-releasing hormone during pregnancy. J. Expo. Sci. Environ. Epidemiol..

[261] Lin C.-Y., Lee H.-L., Hwang Y.-T., Su T.-C. (2020). The association between total serum isomers of per- and polyfluoroalkyl substances, lipid profiles, and the DNA oxidative/nitrative stress biomarkers in middle-aged Taiwanese adults. Environ. Res..

[262] Calloway E.E., Chiappone A.L., Schmitt H.J., Sullivan D., Gerhardstein B., Tucker P.G., Rayman J., Yaroch A.L. (2020). Exploring Community Psychosocial Stress Related to Per- and Poly-Fluoroalkyl Substances (PFAS) Contamination: Lessons Learned from a Qualitative Study. Int. J. Environ. Res. Public Health.

[263] Omoike O.E., Pack R.P., Mamudu H.M., Liu Y., Strasser S., Zheng S., Okoro J., Wang L. (2021). Association between per and polyfluoroalkyl substances and markers of inflammation and oxidative stress. Environ. Res..

[264] Nielsen N.R., Strandberg-Larsen K., Grønbæk M., Kristensen T.S., Schnohr P., Zhang Z.-F. (2007). Self-Reported Stress and Risk of Endometrial Cancer: A Prospective Cohort Study. Psychosom. Med..

[265] Marcus D., King A., Yazbek J., Hughes C., Ghaem-Maghami S. (2021). Anxiety and stress in women with suspected endometrial cancer: Survey and paired observational study. Psycho-Oncology.

[266] Madeddu C., Sanna E., Gramignano G., Tanca L., Cherchi M.C., Mola B., Petrillo M., Macciò A. (2022). Correlation of Leptin, Proinflammatory Cytokines and Oxidative Stress with Tumor Size and Disease Stage of Endometrioid (Type I) Endometrial Cancer and Review of the Underlying Mechanisms. Cancers.

[267] Ferrandina G., Petrillo M., Mantegna G., Fuoco G., Terzano S., Venditti L., Marcellusi A., De Vincenzo R., Scambia G. (2014). Evaluation of quality of life and emotional distress in endometrial cancer patients: A 2-year prospective, longitudinal study. Gynecol. Oncol..

[268] Reid H.W., Broadwater G., de Oca M.K.M., Selvan B., Fayanju O., Havrilesky L.J., Davidson B.A. (2022). Distress screening in endometrial cancer leads to disparity in referral to support services. Gynecol. Oncol..

[269] Heidari F., Rabizadeh S., Mansournia M.A., Mirmiranpoor H., Salehi S.S., Akhavan S., Esteghamati A., Nakhjavani M. (2019). Inflammatory, oxidative stress and anti-oxidative markers in patients with endometrial carcinoma and diabetes. Cytokine.

[270] Wee S.Y., Aris A.Z. (2023). Revisiting the “forever chemicals”, PFOA and PFOS exposure in drinking water. NPJ Clean Water.

[271] Germano M.L., dos Santos Gomes C., de Souza Barbosa J.F., Neto N.J., Pereira D.S., Ahmed T., Borrero C.L.C., Guerra R.O. (2023). Allostatic load and physical performance in older adults: Findings from the International Mobility in Aging Study (IMIAS). Arch. Gerontol. Geriatr..

[272] Irelli A., Ranieri J., Sirufo M.M., De Pietro F., Casalena P., Ginaldi L., Cannita K., Di Giacomo D. (2022). Allostatic Load as an Insight into the Psychological Burden after Primary Treatment in Women with Breast Cancer: Influence of Physical Side Effects and Pain Perception. J. Clin. Med..

[273] Key T.J., Appleby P.N., Reeves G.K., Roddam A.W. (2010). Insulin-like growth factor 1 (IGF1), IGF binding protein 3 (IGFBP3), and breast cancer risk: Pooled individual data analysis of 17 prospective studies. Lancet Oncol..

[274] Ding S., Madu C.O., Lu Y. (2020). The impact of hormonal imbalances associated with obesity on the incidence of endometrial cancer in postmenopausal women. J. Cancer.

[275] Bruno V., Corrado G., Baci D., Chiofalo B., Carosi M.A., Ronchetti L., Piccione E., Albini A., Noonan D.M., Piaggio G. (2020). Endometrial Cancer Immune Escape Mechanisms: Let Us Learn From the Fetal-Maternal Interface. Front. Oncol..

[276] Zhan L., Liu X., Zhang J., Cao Y., Wei B. (2020). Immune disorder in endometrial cancer: Immunosuppressive microenvironment, mechanisms of immune evasion and immunotherapy. Oncol. Lett..

[277] Kamendulis L.M., Hocevar J.M., Stephens M., Sandusky G.E., Hocevar B.A. (2022). Exposure to perfluorooctanoic acid leads to promotion of pancreatic cancer. Carcinogenesis.

[278] Durham J., Tessmann J.W., Deng P., Hennig B., Zaytseva Y.Y. (2023). The role of perfluorooctane sulfonic acid (PFOS) exposure in inflammation of intestinal tissues and intestinal carcinogenesis. Front. Toxicol..

[279] Tyrrell J., Melzer D., Henley W., Galloway T.S., Osborne N.J. (2013). Associations between socioeconomic status and environmental toxicant concentrations in adults in the USA: NHANES 2001–2010. Environ. Int..

[280] Liddie J.M., Schaider L.A., Sunderland E.M. (2023). Sociodemographic Factors Are Associated with the Abundance of PFAS Sources and Detection in US Community Water Systems. Environ. Sci. Technol..

